# Multimodal Analysis of the Tissue Response to a Bone-Anchored Hearing Implant: Presentation of a Two-Year Case Report of a Patient With Recurrent Pain, Inflammation, and Infection, Including a Systematic Literature Review

**DOI:** 10.3389/fcimb.2021.640899

**Published:** 2021-03-30

**Authors:** Martin L. Johansson, Tim G.A. Calon, Omar Omar, Furqan A. Shah, Margarita Trobos, Peter Thomsen, Robert J. Stokroos, Anders Palmquist

**Affiliations:** ^1^ Department of Biomaterials, Institute of Clinical Sciences, Sahlgrenska Academy, University of Gothenburg, Gothenburg, Sweden; ^2^ Research and Technology, Oticon Medical AB, Askim, Sweden; ^3^ Department of Otorhinolaryngology, Head and Neck Surgery, Maastricht University Medical Center, Maastricht, Netherlands; ^4^ Department of Otorhinolaryngology, Head and Neck Surgery, University Medical Center Utrecht, Utrecht, Netherlands

**Keywords:** BAHS, bone-anchored hearing, osseointegration, infection, pain, BAHA (bone anchored hearing aid), Holgers Index, histology

## Abstract

Osseointegration is a well-established concept used in applications including the percutaneous Bone-Anchored Hearing System (BAHS) and auricular rehabilitation. To date, few retrieved implants have been described. A systematic review including cases where percutaneous bone-anchored implants inserted in the temporal bone were retrieved and analyzed was performed. We also present the case of a patient who received a BAHS for mixed hearing loss. After the initial surgery, several episodes of soft tissue inflammation accompanied by pain were observed, leading to elective abutment removal 14 months post-surgery. Two years post-implantation, the implant was removed due to pain and subjected to a multiscale and multimodal analysis: microbial DNA using molecular fingerprinting, gene expression using quantitative real-time polymerase chain reaction (qPCR), X-ray microcomputed tomography (micro-CT), histology, histomorphometry, backscattered scanning electron microscopy (BSE-SEM), Raman spectroscopy, and fluorescence *in situ* hybridization (FISH). Evidence of osseointegration was provided *via* micro-CT, histology, BSE-SEM, and Raman spectroscopy. Polymicrobial colonization in the periabutment area and on the implant, including that with *Staphylococcus aureus* and *Staphylococcus epidermidis*, was determined using a molecular analysis *via* a 16S-23S rDNA interspace [IS]-region-based profiling method (IS-Pro). The histology suggested bacterial colonization in the skin and in the peri-implant bone. FISH confirmed the localization of *S. aureus* and coagulase-negative staphylococci in the skin. Ten articles (54 implants, 47 patients) met the inclusion criteria for the literature search. The analyzed samples were either BAHS (35 implants) or bone-anchored aural epitheses (19 implants) *in situ* between 2 weeks and 8 years. The main reasons for elective removal were nonuse/changes in treatment, pain, or skin reactions. Most samples were evaluated using histology, demonstrating osseointegration, but with the absence of bone under the implants’ proximal flange. Taken together, the literature and this case report show clear evidence of osseointegration, despite prominent complications. Nevertheless, despite implant osseointegration, chronic pain related to the BAHS may be associated with a chronic bacterial infection and raised inflammatory response in the absence of macroscopic signs of infection. It is suggested that a multimodal analysis of peri-implant health provides possibilities for device improvements and to guide diagnostic and therapeutic strategies to alleviate the impact of complications.

## Introduction

Following the development of titanium implants for oral rehabilitation in 1977, the bone-anchored hearing system (BAHS) became an established form of hearing rehabilitation for subjects suffering from conductive or mixed hearing loss and single-sided deafness (Snik et al., 2005). The system consists of a screw-shaped implant inserted in the temporal bone and mounted with a percutaneous abutment, onto which a sound processor is attached. It relies on permanent fixation of the implant through osseointegration, and is overall considered to be a successful treatment option with good clinical outcomes (Lagerkvist et al., 2020). The complications are mainly related to inflammation and infection of the soft tissue surrounding the abutment, pain, loss of skin sensibility, and implant loss (Verheij et al., 2016).

The success rates for permanently implanted biomaterials are generally very high. On rare occasions, however, implants may need to be explanted. In addition to elective causes (e.g., pain, discomfort, psychological and esthetic considerations), reasons for the retrieval of bone-anchored clinical implants include mechanical failures and peri-implant infections. Retrievals of percutaneous osseointegrated limb prostheses have been shown to provide an opportunity to evaluate the intact bone-implant interface (Palmquist et al., 2008; Palmquist et al., 2014), as well as the abutment-soft tissue interface (Lennerås et al., 2017; Trobos et al., 2018).

Preclinical studies of implants are vital for translation into clinical applications. These studies typically include *in vitro* testing and subsequent *in vivo* implantation of functional devices in an appropriate animal model. However, relative to clinical studies, animal models rarely fully mimic relevant human *in vivo* situations in terms of anatomy, physiology, and pathology. Moreover, the clinical performance of bone-anchored percutaneous implants is typically assessed by subjective clinical assessments and methods to evaluate the stability of the implant-bone unit. Inevitably, the retrieval and evaluation of implants and associated tissues from humans will provide important knowledge of the tissue response to the device in different tissue compartments. By ensuring that the implant is retrieved with the associated biological tissues, information pertaining to the causes of adverse reactions, failure mechanisms, and factors contributing to successful tissue integration can thus be obtained (Palmquist et al., 2014; Lennerås et al., 2017; Trobos et al., 2018). The information gained (along with clinical data) may serve to inform device improvements, understand and eliminate complications, enable appropriate diagnostic procedures and guide therapeutic management strategies in order to alleviate the impact of complications.

Elective removal of BAHS devices is relatively rare. Therefore, to date few retrieved BAHS implants have been investigated (van der Pouw et al., 1998; Bolind et al., 2000; Granström, 2000; Mylanus et al., 2002; Mlynski et al., 2008; Monksfield et al., 2011). Moreover, the technique of determining the status of the tissue adjacent to retrieved BAHS has largely been limited to histology on thick sections.

Chronic or recurrent pain after BAHS implantation, for no apparent reason, affects a small percentage of the treated population. Here we present a case of idiopathic chronic pain after BAHS implantation. In this study we aim to provide a multimodal explorative analysis to enhance the understanding of this important clinical question. The introduction and application of new analytical techniques is an important complement to the data obtained in clinical trials since it allows the exploration of the biological events in various peri-implant tissue compartments. The case is presented according to the CARE Guidelines (Riley et al., 2017), and provides a detailed analysis of a patient with recurrent adverse soft tissue reactions and pain, eventually leading to elective removal of the abutment and implant. The patient in this case report was enrolled in a larger clinical trial (Calon et al., 2018a; Strijbos et al., 2021) where data obtained in the trial, together with analyzes justified by the explant analysis, is presented. Techniques including analysis of microbial DNA using molecular fingerprinting, gene expression using quantitative real-time polymerase chain reaction (qPCR), X-ray microcomputed tomography (micro-CT), histology, histomorphometry, backscattered electron scanning electron microscopy (BSE-SEM), Raman spectroscopy, and fluorescence *in situ* hybridization (FISH) are employed. In addition, a systematic literature review is performed following the PRISMA-P checklist (Moher et al., 2015) to identify and appraise previously published analyses of retrieved BAHS implants and abutments. Finally, to further improve the understanding of osseointegration and adverse events in BAHS, a suggested approach for analysis of retrieved implant and tissue samples is provided.

## Materials and Methods

### Case Report

#### Implant System

The BAHS implant system consists of an abutment (5 mm in diameter) and a screw-shaped implant (4 mm long and 4.5 mm in diameter) machined from commercially pure titanium (Ti) grade 4 (Ponto System, Oticon Medical AB, Askim, Sweden). After machining, the resulting surfaces have average surface roughness values (Sa) of 0.17 and 0.27 µm for the abutment and implant, respectively. (Shah et al., 2016; Trobos et al., 2018) The corresponding values for the developed surface ratio (Sdr) are 82.0% and 14.4%, respectively.

#### Case Presentation

A 39-year-old Caucasian female was treated for mixed hearing loss of the left ear by receiving a 4-mm Ponto Wide implant mounted with a 12-mm abutment (Oticon Medical, Askim, Sweden) in February 2015. The patient was enrolled in a multicenter, randomized, and controlled trial comparing two different techniques for installing the BAHS: the Minimally Invasive Ponto Surgery (MIPS) technique and the linear incision technique with soft tissue preservation (Calon et al., 2016; Calon et al., 2018a; Strijbos et al., 2021). The patient underwent implant surgery using the Minimally Invasive Ponto Surgery, MIPS (Oticon Medical) through a punch technique (Calon et al., 2016; Johansson et al., 2017). Her otological history included several ear surgeries leading to the creation of a radical cavity on her left side. Her medical history included well-controlled type-2 diabetes treated with liraglutide (Victoza, Novo Nordisk, Denmark) and metformin since 2008 and 2010, respectively. High cholesterol was treated with Crestor (AstraZeneca, United Kingdom).

**Figure 1:**
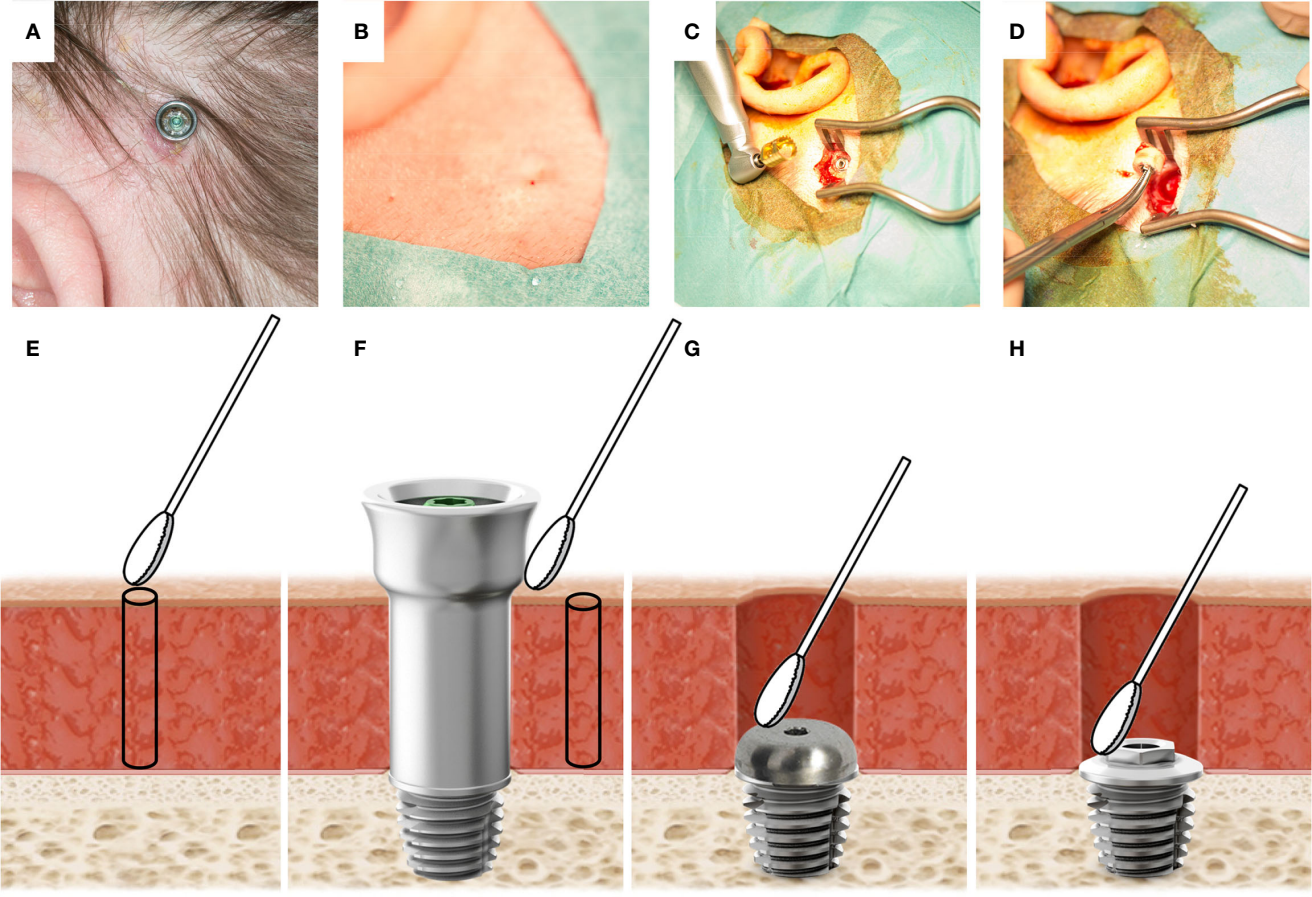
**(A)** The BAHS with a mounted abutment at the 12-week follow-up. In the lower-left quadrant, a reddish moist irritated site can be observed. **(B)** The skin after abutment removal. No signs of inflammation are observed. **(C)** The implant in the bone during removal surgery. On the left: Trephine used to remove the implant is shown. **(D)** Retrieved implant with the surrounding bone. **(E)** Illustration of sampling performed at baseline prior to implantation where a skin swab (2x2 cm) is taken at the intended implantation site, prior to the retrieval of a 1 mm soft tissue biopsy using a 1 mm biopsy punch, **(F)** at 12 weeks, and during episodes with adverse skin reactions, a swab from the external side of the abutment and the peri-abutment skin are taken, and a 1 mm skin biopsy is retrieved. Finally, at implant removal, swabs were taken on the cover screw **(G)** and the top of the implant **(H)**.

After successful implantation, the primary implant stability quotient (ISQ) was determined to be 51 (Ostell ISQ equipment, Ostell AB, Gothenburg, Sweden). The ISQ value rose during the follow-up and remained stable at 57 up to a year following implantation (**Table 1**). The local, macroscopic status of the skin surrounding the percutaneous abutment was assessed using the Holgers Index (Holgers et al., 1988). During follow-up, the patient experienced adverse soft-tissue reactions with two episodes of Holgers score 2 (at 6 and 11 months) and one with a registered Holgers score of 3 (at 14 months) (**Table 1**, **Figure 1A**). In an attempt to reduce adverse reactions, first, treatment with topical antibiotic cream (Terra-Cortril, Pfizer, New York, NY, United States) was applied, and finally the abutment was removed in May 2016, 15 months after initial insertion, leaving the implant in the bone for possible future reinstallation of an abutment. After removal of the abutment, the skin healed, closing the wound over the implant without macroscopic signs of inflammation (**Figure 1B**). However, the patient continued to experience episodes of pain leading to elective implant removal surgery in March 2017, 25 months after implantation (**Figures 1C, D**).

**Table 1:** Clinical outcomes, samplings, and treatments at the visits.

Visit No.	Time^a)^	Reason for visit	Holgers^b)^	ISQ High	ISQ Low	Pain around implant^c)^	Radiating pain^c)^	Headache^c)^	Biopsy	Swab	Treatment, medication and comments
1	0d	Surgery	–	52	51	–	–	–	Yes	Yes	Terra-Cortril on ribbon for nine days with healing cap.
2	9d	Standard follow-up visit	0	55	53	1	0	0	No	No	Wound dehiscence, therefore Terra-Cortril ointment.
3	20d	Standard follow-up visit	0	53	53	0	0	0	No	No	Slight dehiscence.
4	24d	Pain after cleaning of abutment	0	53	52	6	0	1	No	No	Paracetamol if needed.
5	12w	Standard follow-up visit	1	–	–	0	0	0	Yes	Yes	Crust formation. Uses Terra-Cortril ointment 2 times per week.
6	6.3m	Pain around abutment	2	57	56	2	0	3	Yes	Yes	Terra-Cortril ointment (3 times per day for 1 week).
7	6.7m	Check after Holgers 2	1	56	56	0	0	0	No	Yes	Skin better. IS-PRO after Terra-Cortril treatment.
8	11m	Pain after wearing sound processor and skin irritation	2	58	57	1	0	5	No	Yes	Continuous inflammation under Terra-Cortril use. Revision surgery planned.
9	11.7m	Revision surgery under local anaesthesia	–	57	57	–	–	–	No	No	Terra-Cortril on ribbon with healing cap (1 week).
10	12m	Check-up after revision	0	57	57	0	0	2	No	No	Terra-Cortril ointment (1 time per day 1 week).
11	13.6m	Standard follow-up visit	1	57	57	1	0	2	No	No	–
12	14.3m	Irritation complaints	3	57	57	2	3	0	No	Yes	Removal of abutment with placement of cover screw.
13	15m	Check-up 2 weeks after abutment removal	–	–	–	0	0	0	No	No	Pain after abutment removal lasting 1.5 weeks.
14	25.5m	Implant removal surgery	–	–	–	–	–	–	No	Yes	Implant explantation

^a)^Days(d), weeks (w) or months (m) after surgery.

^b)^Holgers Index: 0 No irritation; 1 Slight redness; 2 Red and slightly moist tissue, no granuloma formation; 3 Reddish and moist; sometimes granulation tissue; 4 Removal of skin-penetrating implant necessary due to infection.

^c)^Pain scores is graded in a 10-point scale. 0 representing “no pain” to 10 representing “the worst pain imaginable”.

- Values not obtained or not available.

#### Ethics

The study was approved by the medical ethics committee (METC, azM/UM, Maastricht, The Netherlands) (NL50072.068.14). Additional consent was provided by the responsible ethics committee at MUMC+ for the retrieval of the BAHS implant. The patient provided additional written informed consent for this case study.

#### Clinical Assessment

During the study, clinical assessment and sampling were performed according to the study protocol (Calon et al., 2016). The patient was assessed at inclusion, surgery, standard follow-up visits (9 days, 3 and 12 weeks), and extra consultations. During all visits, the peri-abutment skin condition was graded using the Holgers Index (a five-grade scale where: 0 No irritation; 1 Slight redness; 2 Red and slightly moist tissue, no granuloma formation; 3 Reddish and moist; sometimes granulation tissue; and 4 Removal of skin-penetrating implant necessary due to infection) (Holgers et al., 1988). Skin reactions assessed as Holgers ≥ 2 were defined as an incidence of inflammation. Furthermore, pain scores indicated by the patient, with 0 indicating no pain at all and 10 indicating the worst conceivable pain, were obtained at all visits. Skin sensibility and wound healing were assessed at standard follow-up visits, and the presence of skin dehiscence and sagging, soft-tissue height, soft-tissue overgrowth, and processors use were assessed at all follow-up visits. Some parameters were also assessed at extra visits. The Implant Stability Quotient (ISQ) (Osstell) measurements were obtained directly after surgery and at all follow-up visits.

#### Sample Collection

A baseline swab for microbiological analysis was obtained from a 2 by 2 cm area at the intended implant site after shaving the hair but before disinfecting the area. After surgery, additional bacterial swab samples were obtained from the peri-abutment skin site at 12 weeks, as well as during episodes of adverse soft-tissue reactions (Holgers score ≥ 2) (**Table 1**). The available external side of the abutment and approximately 1 cm of peri-abutment skin were swabbed. If peri-abutment fluid (e.g., moist) was present at the peri-abutment skin, this was obtained using the same swab. No cleaning or disinfection was performed before sampling. The samples were stored in an Eppendorf container with 200 μL of transport buffer (IS-Diagnostics Ltd, Amsterdam) at -20°C.

The 5 mm skin punch removed during primary surgery was collected for baseline gene expression. At the 12-week follow-up and during episodes of inflammation, 1 mm soft tissue biopsies (Integra, York, USA) were obtained close to the abutment from patients who volunteered. Samples were snap-frozen in liquid nitrogen and stored at -80°C for a subsequent molecular analysis using qPCR (**Table 1**, **Figures 1E, F**).

#### Implant Removal

At the time of implant removal, a 5-mm biopsy of the soft tissue above the submerged implant was retrieved and immersed in a 4% paraformaldehyde solution, and bacterial swabs were obtained from the cover screw and the top of the implant (**Figures 1G, H**). The implant and the surrounding bone were retrieved *en bloc* using a trephine and were immediately submerged in a 4% paraformaldehyde solution. The soft tissue biopsy and the implant were thereafter transported to the Department of Biomaterials at the University of Gothenburg (Gothenburg, Sweden), where it was entered into the biobank (Biobank ID 513). Following dehydration in a graded series of ethanol, the implant-bone sample was resin embedded in LR White (London Resin Co. Ltd, UK), while the soft tissue biopsy was embedded in paraffin according to standard methods.

#### Microbiology

The bacterial samples were processed according to the IS-pro technique, which is a bacterial profiling technique that can detect bacterial species at the DNA level (Budding et al., 2010; Budding et al., 2016; de Meij et al., 2016). This technique has been validated against 16S rRNA Next-Generation-Sequencing. The procedures have previously been described (Budding et al., 2010; Rutten et al., 2015; de Meij et al., 2016; Calon et al., 2019; Cranendonk et al., 2019). The IS-pro™ technique is based on the categorization of species-specific length differences in the 16S-23S rRNA gene interspacer region of bacteria. This region is located at the end of 16S and the beginning of 23S, which can vary between 200 and 2000 base pairs (bp) in length. In short, the samples were stored in an Eppendorf container with 200 µL of the Transportbuffer (IS-Diagnostics, Amsterdam, Netherlands). The DNA was extracted using an easyMAG machine (bioMerieux Clinical Diagnostics, Marcy-l’ Etoile, France). Isolated DNA was processed according to the IS-pro™ assay (IS-Diagnostics, Amsterdam, Netherlands). Two standardized multiplex PCR amplifications were performed. The first PCR is specific for Firmicutes, Actinobacteria, Fusobacteria, Verrucomicrobia, and Bacteroidetes (FAFV). The second PCR is specific for Proteobacteria. PCR amplifications were performed on a GeneAmp PCR system 9700 (Applied Biosystems, Foster City, CA, United States). PCR fragment separation was performed on an ABI Prism 3500 genetic analyzer (Applied Biosystems). The resulting profiles consist of peaks with a specific length and height. These profiles were analyzed for species identification and quantification using IS-pro software (IS-Diagnostics, Amsterdam, Netherlands). The data were visualized using Spotfire version 7.10 ITIBCO, Palo Alto, CA, United States) and R version 3.3.2. (R Foundation for Statistical Computing, Vienna, Austria).

#### RNA Extraction and Quantitative Real-Time Polymerase Chain Reaction (q-PCR)

The methods for q-PCR have been previously described, and the results for subjects included in the multicenter trial have been published (Calon et al., 2018c). In short, cDNA was obtained by isolating RNA using TRI Reagent (Sigma, St. Louis, MO, USA). Approximately 750 ng of RNA was transcribed to cDNA using the SensiFast cDNA Synthesis Kit. The expressions of genes related to inflammation (IL-1ß, IL-6, TNF-α, TGF-ß, MIP-1α), tissue metabolism (TIMP-1, COL1α1), vascularization (VEGF, FGF-2), and bacterial infection (TLR-2, TRL-4) (Sigma-Aldrich, St. Louis, Missouri, USA) were quantified and determined. The relative expressions were determined using LinRegPCR (version 2016.1).

#### Micro-CT

The intact resin-embedded specimen was scanned in a Skyscan 1172 (Bruker micro-CT, Kontich, Belgium) micro-CT system operating at 100 kV. The resolution was set to 5.88 µm, with 5 images averaging each 0.4° rotation step. The projection images were reconstructed *via* back-projection, manually aligned along the long axis of the implant, evaluated in terms of bone growth in the threaded volume, and visualized in the associated program suite (NRecon, Dataviewer, CTAn, CTVox, and CTVol). In brief, a volume of interest was defined as a tapered cylinder encompassing the threaded area of the implant, wherein the bone volume was segmented *via* manual global thresholding based on the morphology (Palmquist et al., 2017), prior to performing a 3D analysis. The binary, segmented images of the implant, regions of interest, and bone within the regions of interest were saved. A section matching the histological ground section was found by the manual alignment of the saved segmented dataset, and the 3D segmentation was directly compared to the histomorphometry by a 2D analysis of the matching section.

#### Histology and Histomorphometry

Following micro-CT scanning, 50 µm thick central ground sections were prepared by sawing and grinding (EXAKT^®^ Apparatebau GmbH & Co, Norderstedt, Germany) (Donath and Breuner, 1982) and subsequently stained with toluidine blue or May-Grünewald Giemsa staining. Qualitative histology and quantitative histomorphometry were performed to determine the amount of bone-to-implant contact (BIC) and bone area (BA) within the implant threads using light optical microscopy (Nikon Eclipse E600, Nikon NIS-Elements software, Nikon Instruments Europe BV, Amsterdam, The Netherlands).

#### Backscattered Electron Scanning Electron Microscopy (BSE-SEM)

The remaining resin-embedded bone-implant block was wet polished with 400–4000 grit SiC grinding paper. The samples were air-dried overnight prior to low-vacuum BSE-SEM imaging in a Quanta 200 environmental SEM (FEI Europe B. V, Eindhoven, The Netherlands), operated at 20 kV and a 0.5 Torr water vapor pressure.

#### Micro-Raman Spectroscopy

Raman imaging was performed using a confocal Raman microscope (WITec alpha300 R, Ulm, Germany) equipped with a 532 nm laser, as previously described (Shah et al., 2017; Shah et al., 2018). Briefly, the laser was focused down onto the sample surface using a ×10 objective with a numerical aperture of 0.25. The spectra were collected in the 300–1800 cm^-1^ spectral range using an electron-multiplying charge-coupled device (EMCCD) detector cooled to -60°C behind a 600 mm^-1^ grating at a spectral resolution of ~4 cm^-1^, with an integration time of 2 s per pixel, and a pixel size of 2 µm × 2 µm.

#### Fluorescence in Situ Hybridization (FISH)

Following embedding and sectioning of the soft tissue biopsy obtained at explantation, fluorescence *in situ* hybridization (FISH) was performed using a *Staphylococcus aureus-*CoNS specific peptide nucleic acid (PNA) probe kit (KT005, AdvanDx A/S, Vedbæk, Denmark) for the direct identification and localization of *S. aureus* (as green) and coagulase-negative staphylococci (CoNS) (as red) in the tissue. A drop of PNA probe in the hybridization solution was added to each tissue section, a coverslip was added, and the slides were placed in a hybridization oven at 55°C for 90 min. The slides were subsequently washed in a wash solution at 55°C for 30 min and air-dried. The mounting medium and a coverslip were applied, and the stained slides were visualized under a fluorescence microscope (Eclipse E600, Nikon Instruments Europe BV) and a confocal microscope (C2plus, Nikon Instruments Europe BV) with a plan-apochromat 60x/1.2 water immersion objective. The excitation/emission spectra used were a blue filter (340-380/435-485 nm), a green filter (465-495/515-555 nm), and a red filter (540-580/600-660 nm).

### Systematic Literature Review

A systematic review was undertaken according to the Preferred Reporting Items for Systematic reviews and Meta-Analysis Protocols (PRISMA-P) checklist (Moher et al., 2015) (**Supplementary 1**).

#### Eligibility Criteria

Inclusion criteria included analyses of lost or electively removed percutaneous bone anchored implants inserted in the temporal bone using a systematic approach (**Table 2**). Any report, irrespective of language, analytical methods, study type, patient, and without the restriction of time of publication were included. Only articles reporting the analysis of retrieved implants, including surrounding bone, and with or without the abutment attached were included. Bone-anchored hearing systems (BAHSs) and percutaneous bone anchored implants for auricular epithesis (BAAEs) were included. Articles (or part of articles) reporting analyses of other types of extraoral implants not placed in the temporal bone were excluded.

**Table 2:** Inclusion and exclusion criteria.

Language	Any language
Population	Human subject of any age with bone-anchored hearing systems (BAHSs) or percutaneous bone anchored implants for auricular epithesis (BAAEs) spontaneously lost or requiring explantation
Intervention	Removal of BAHS or BAAE
Comparison	Findings from analysis
Reported outcome	Any type of reported outcome subsequent analysis of retrieved implants and tissue samples (e.g., histology, histomorphometry, microbiology)
Study design	Any type of human study and case reports
Time frame	Article published up to September 9, 2020
Exclusion criteria	Publication not fulfilling the eligibility criteria. In vitro and animal experiments, expert opinions and communications were all excluded. Extra-oral osseointegrated implants not placed in the temporal bone region (e.g., implants for auricular epithesis retention) were eliminated from the analysis.

#### Search Strategy

The systematic literature search was performed in MEDLINE, SCOPUS, and ISI Web of Science for articles published anytime up to September 9, 2020. The electronic search was conducted using the following string: (((bone-anchored hearing) OR (bone anchored auricular) OR (percutaneous AND bone-anchored) OR BAHS OR BAHA OR BAHI) AND (retrieved OR retrieval OR Histology OR histological OR explanted)) NOT ((al-BAHA) OR (Baha[Author]) OR (BAHS [Author]) OR (Bahi[Author])). This strategy was thereafter adapted to the other databases using the search string according to **Supplementary 2**. This search was complemented by manual search of article database (MLJ) and the citation lists of the included articles to capture any articles not initially identified.

#### Data Analysis

**Figure 2:**
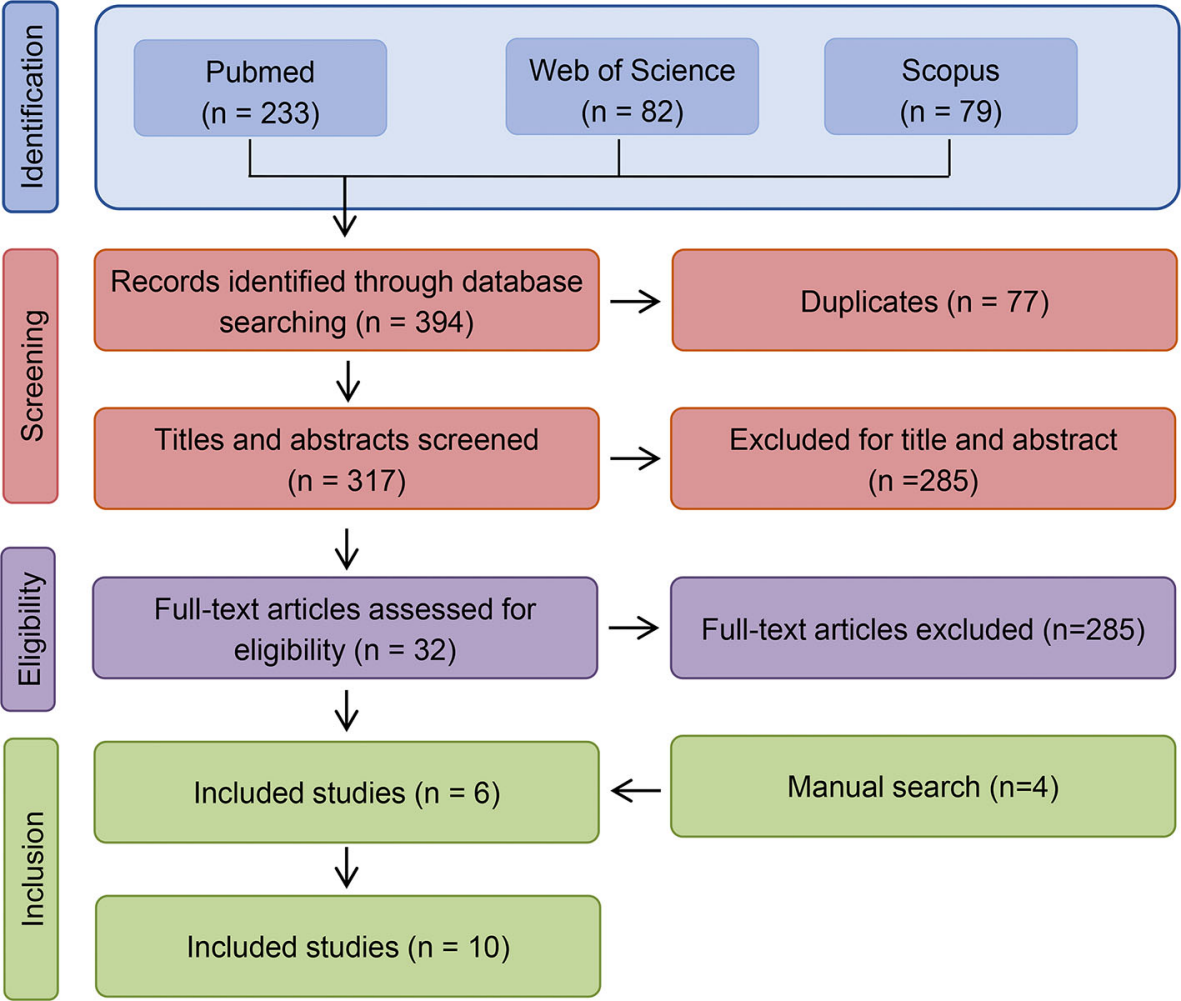
PRISMA flow diagram of search strategy and results.

The initial list of references (n = 394) was independently screened by two authors (MJ and AP), and any discrepancies were resolved *via* consensus. The selection process is described in the PRISMA-P flow chart (**Figure 2**). The two reviewers then independently screened the full text of articles included after abstract review, to assess eligibility. Reasons for excluding trials were recorded, and any disagreements were resolved through consensus. Of note, patients within these articles that did not meet the inclusion criteria above were also excluded (i.e., percutaneous bone anchored implants placed in regions other than the temporal bone). Any disagreements were resolved by discussion among the authors.

Data from the included articles were extracted and entered into Microsoft Excel (Redmond, WA, USA). The type of study, sex, age, number of patients and implants, type and model of the implant (if known), time *in situ*, reason for removal, techniques used for analysis, and main outcome of the analysis were recorded.

## Results

### Case report

#### Microbiological Identification

**Figure 3:**
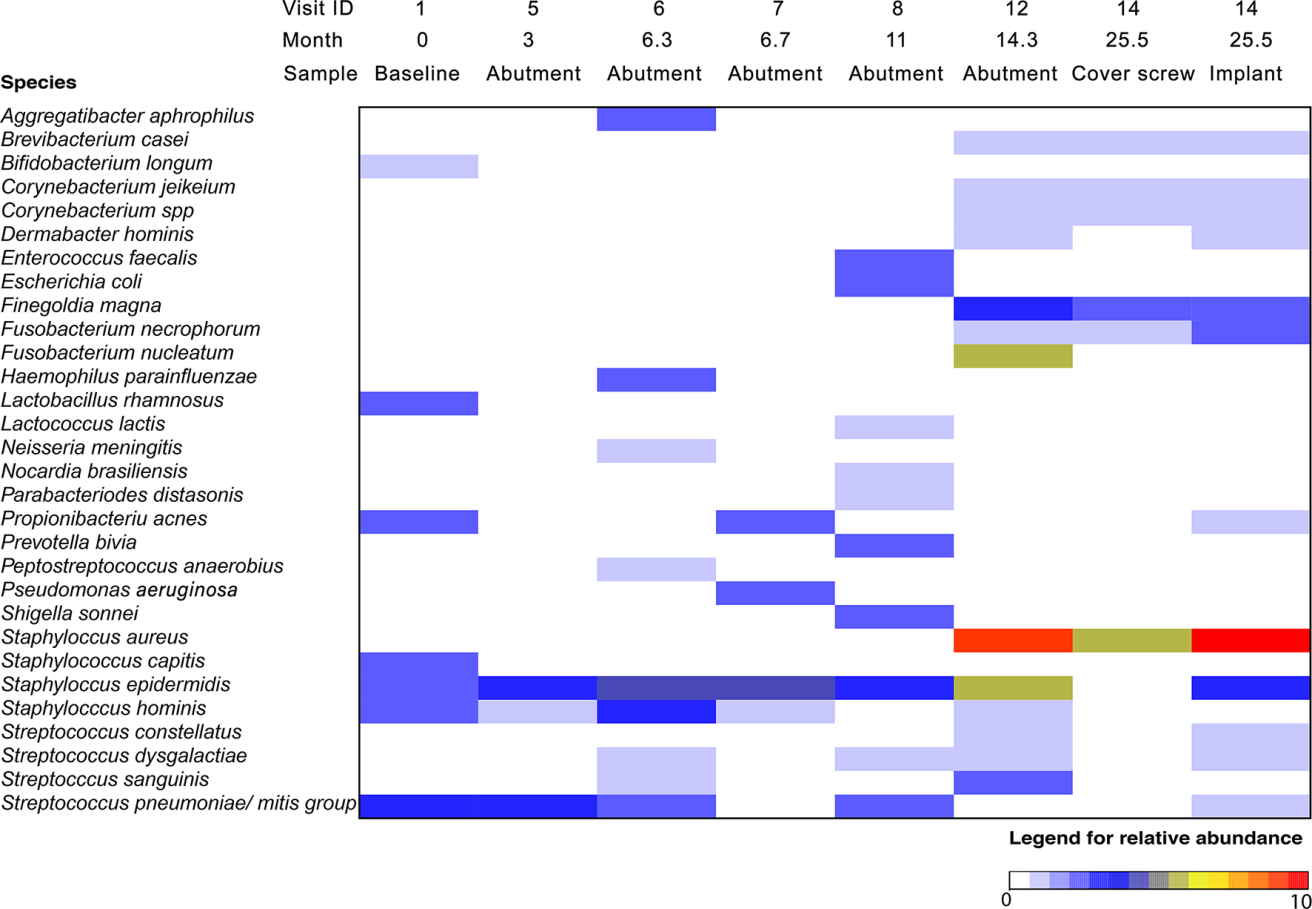
Relative abundance of bacteria on BAHS over time using the IS-pro technique. The color indicates the relative abundance of specific bacterial species at each time point, with red indicating the highest abundance. Location indicates the area where a cotton swab was taken for microbiological analysis: at the baseline, a swab of the intended implantation site was obtained; at different visits, a swab of the skin penetrating abutment and 1 cm of peri-abutment skin was obtained; and at implant retrieval, two additional swabs were obtained from the implant and cover screw.

The IS-pro™ analyses are presented in **Figure 3**. The analyses showed that at baseline, mainly skin bacteria were present, such as *Propionibacterium acnes*, *Staphylococcus capitis*, *Staphylococcus epidermidis*, and *Streptococcus pneumoniae/mitis*. At the 12-week follow-up, only *S. epidermidis*, *Staphylococcus hominis*, and *S. pneumoniae/mitis* were detected. In the following months, two episodes of inflammation were observed. During these episodes of inflammation, more bacterial species were observed, including *Haemophilus parainfluenza*, *Escherichia coli*, and *Enterococcus faecalis* (**Figure 3**). During visit 12, the abutment was removed. Compared to previous visits, a polymicrobial flora, including high amounts of *Staphylococcus aureus* and *Finegoldia magna*, was observed (**Figure 3**). During visit 14, a similar polymicrobial flora was found on both the cover screw and the implant with high quantities of *S. aureus.*

#### Molecular Profile

**Figure 4:**
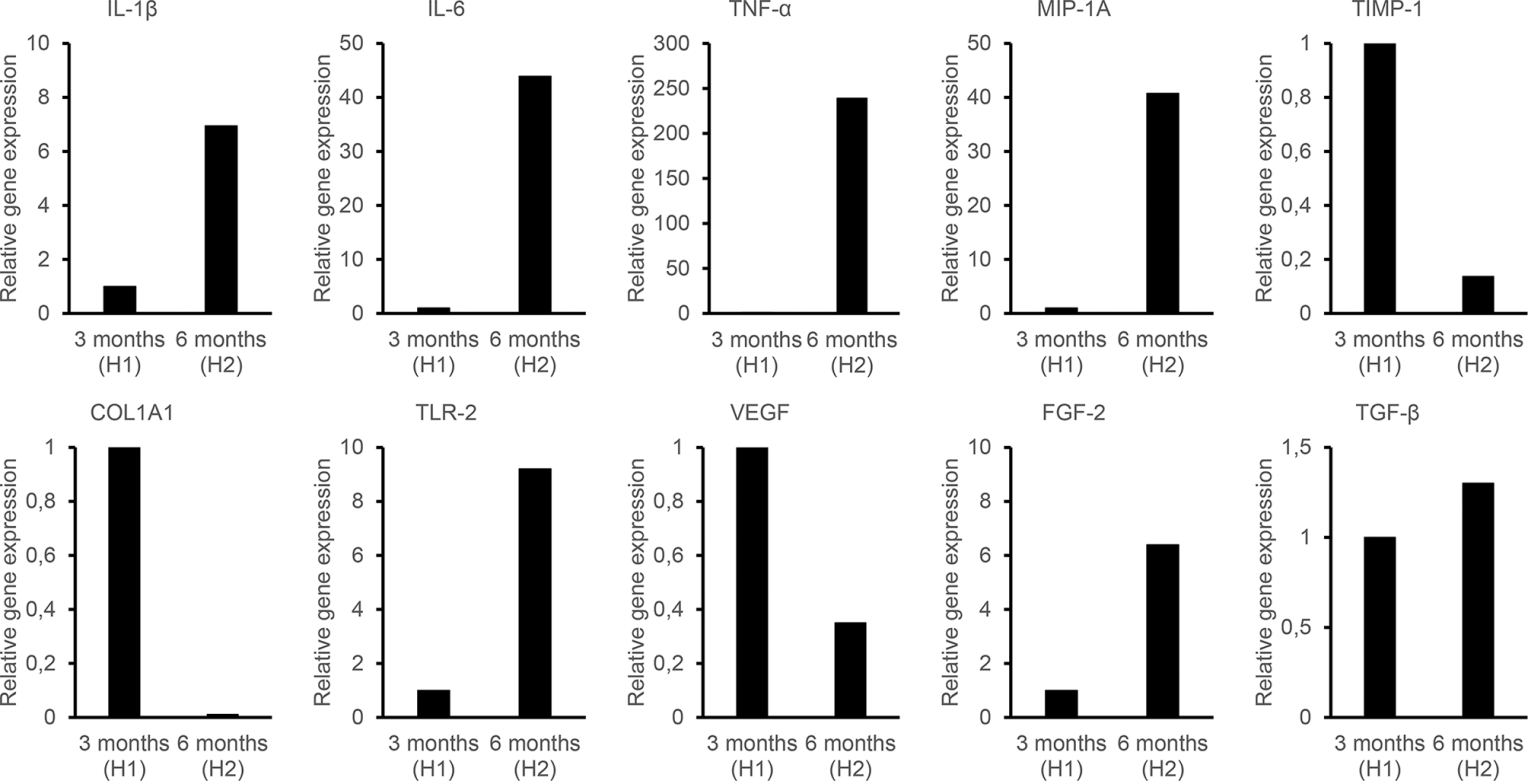
Quantitative PCR analysis to evaluate the relative expression of genes related to inflammation (IL-1b, IL-6, IL-8, TNF-a, IL-17, IL-10, TGF-ß, MIP-1a), extracellular matrix (MMP-9, TIMP-1, COL1a1), vascularization (VEGF, FGF-2), and bacterial infection (TLR-2). H1 indicates the Holgers 1 score according to the Holgers Index scoring. H2 indicates the Holgers 2 score, which was defined as an episode of soft tissue inflammation.

The gene expression levels were determined during a noninflamed state (12 weeks) and an episode of inflammation and pain (6 months) (**Figure 4**). During inflammation, the expression of IL-1β, IL-6, TNF-α, MIP-1α, FGF-2, and TLR-2 in the soft tissue surrounding the abutment was strongly upregulated compared with that in the non-inflamed state at 12 weeks, whereas the expression of TGF-β was only moderately increased. In contrast, the TIMP-1, COL1α1 and VEGF expressions were downregulated during inflammation compared with the 12-weeks expression. No expression of TLR-4 was detected at the baseline or follow-up in any of the samples (baseline, 3 months and inflammation).

#### Micro-CT and Histomorphometry

**Figure 5:**
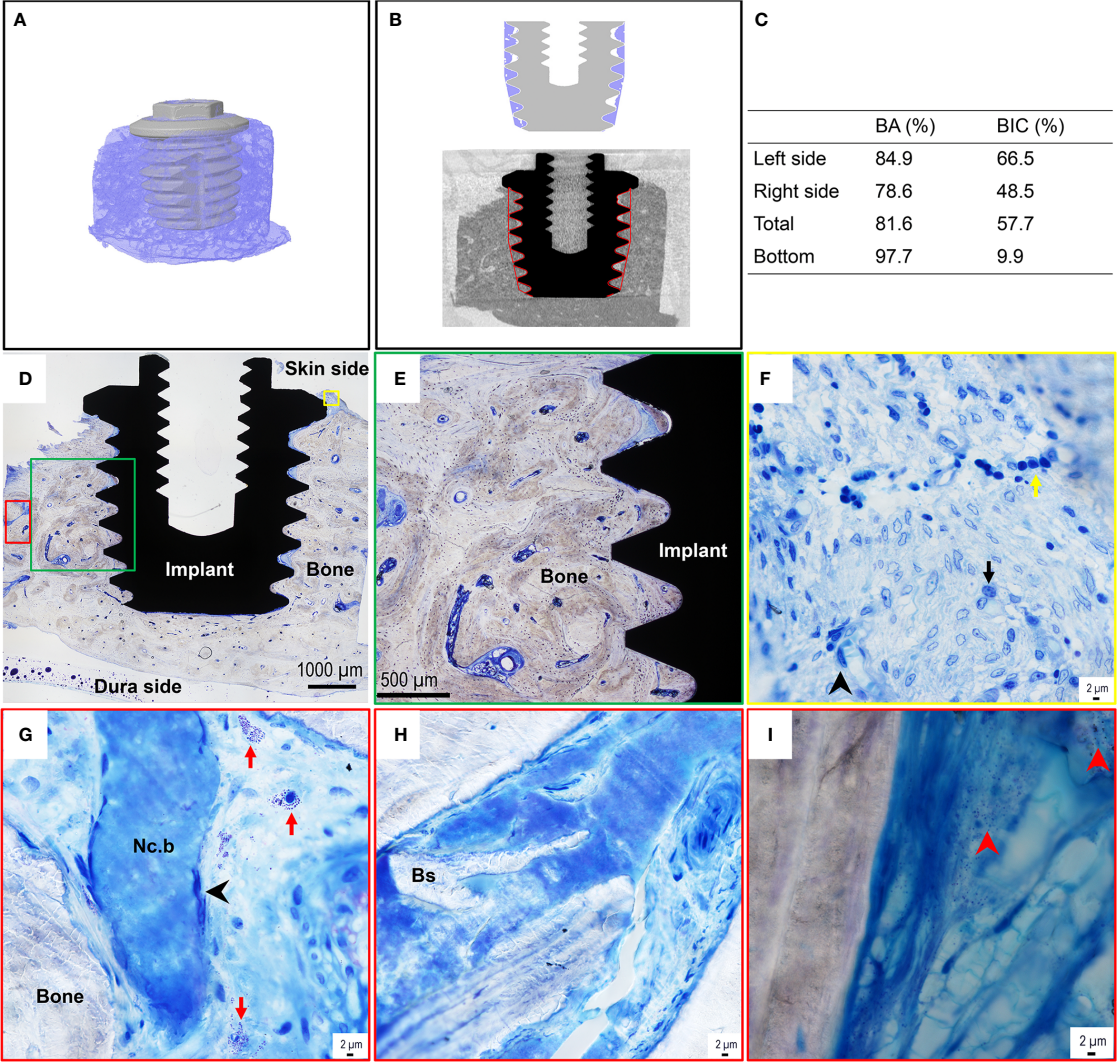
Micro-CT images, histomorphometry, and histological assessment of the bone-implant sample of the BAHS implant. **(A)** MicroCT 3D reconstruction of the retrieved implant with surrounding bone. **(B)** Region of interest and segmentation of the bone surrounding the implant. **(C)** Histomorphometric data, BA=Bone area, BIC=Bone to implant contact. **(D)** Toluidine blue-stained section shows the implant integrated with the recipient temporal bone site, with dense mature bone filling the implant threads and in contact with the titanium surface in most of the threads. The green, yellow and red boxes in **(D)** represent selected regions presented at higher magnifications in **(E–I)**, respectively. **(F)** Toluidine blue-stained sections at high magnification show an inflammatory infiltrate in the top region at the interface of bone with the top flange of the implant. The inflammatory infiltrate consists of mainly chronic inflammatory cells, containing mononuclear/macrophage, lymphocyte, and plasma cell types (exemplified by the black arrowhead, black arrow, and yellow arrow in **(F)**, respectively). **(G, H)** On several occasions, necrotic bone (Nc.b) and bone sequesters (Bs) were found in cavities of the surrounding bone. The necrotic bone islets always appeared surrounded by spindle-shaped elongated macrophages [exemplified by the black arrowhead in **(G)**]. Mast cells were frequently detected in the bone cavities [some are indicated by the red arrow in **(G)**]. **(I)** The Giemsa-stained section shows the presence of coccoid bacteria-like microstructures (exemplified by the red arrowheads) in some cavities of the surrounding bone.

The micro-CT revealed large amounts of bone around the implant, mainly of the cortical type, with only a smaller amount of porosity in the trephined volume (**Figure 5A**). Some fractures originating from the retrieval were visible both close to the implant top and closer to the dura side. A lack of bone tissue was observed close to the implant in 6 out of 13 threads, especially in the thread valley region (**Figure 5B**). The 3D quantification of tissue ingrowth in the threaded volume of the implant showed a bone volume fraction of 86.4%. The segmentation was further compared to the histological measurement, revealing similar values for the 2D sections. The values were 80.8% and 81.6% for the micro-CT and histomorphometry, respectively, thereby validating the 3D results (**Figures 5B, C**). The histomorphometry revealed a direct bone-implant contact (BIC) of 57.7% and a bone area within the threads (BA) of 81.6% (**Figure 5C**).

#### Histological Evaluation of the Bone-Implant Sample

The qualitative histological assessment of the toluidine blue-stained sections revealed that the implant was well integrated in dense, mature, recipient bone (**Figures 5D, E**). In general, the bone appeared filling all the threads of the implant and on many occasions in direct contact with the titanium surface at the light microscopy level. In some threads, the soft tissue separated the implant surface from the surrounding bone in the interface.

At high magnification (×60 water immersion objective), the top region of the bone close to the implant flange revealed a considerable amount of inflammatory infiltrate, containing mainly chronic inflammatory cells, including monocytes/macrophages, lymphocytes, and plasma cells (**Figure 5F**). Whereas few polymorphonuclear cells (PMNs) were observed, mast cells and degranulating mast cells were found on several occasions in the cavities in the surrounding bone (**Figure 5G**). Furthermore, on some occasions small areas of necrotic bone spicules surrounded by macrophages (**Figure 5G**) and bone sequesters (**Figure 5H**) were detected within the surrounding bone at a distance from the implant. Moreover, the evaluation of the Giemsa-stained sections showed the presence of darkly stained bacteria-like microstructures, sporadically found in some of the bone cavities at a distance from the implant (**Figure 5I**).

**Figure 6:**
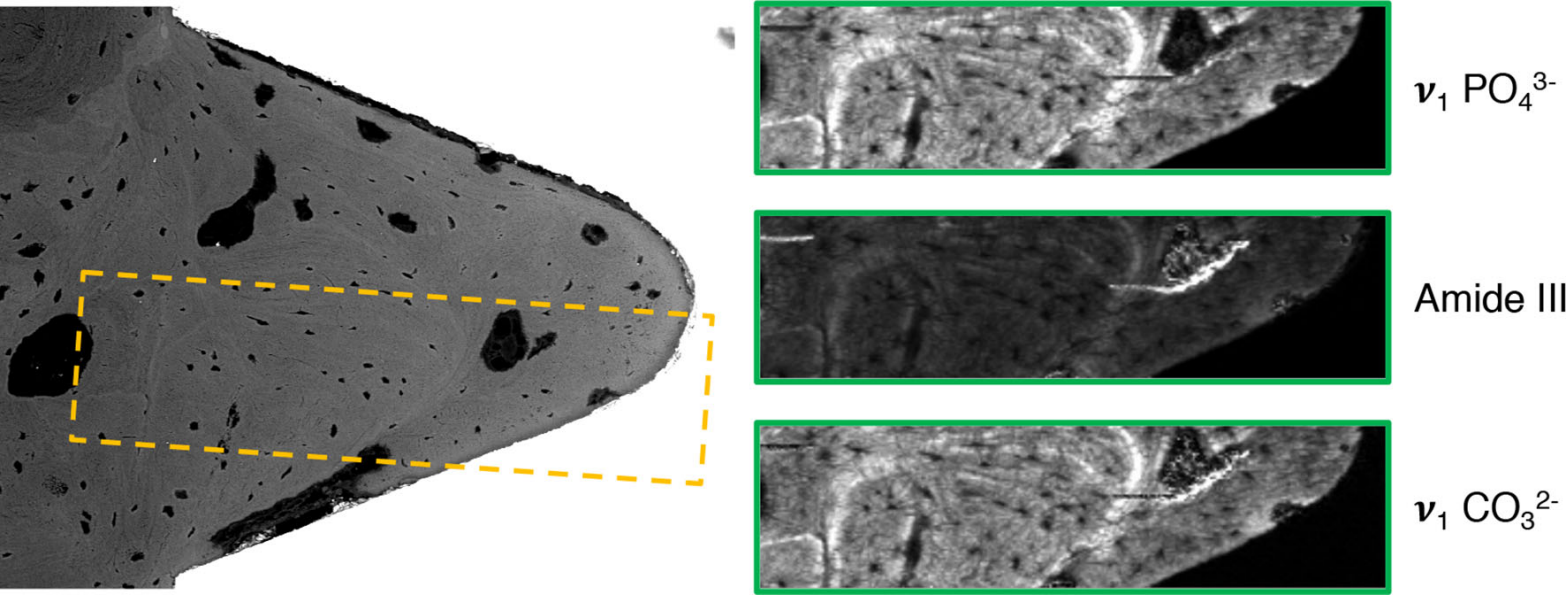
Backscattered electron scanning electron microscopy (BSE-SEM) and Raman imaging corresponding to the yellow box, showing the distribution of phosphate (v_1_ $PO_{4}^{3-}$), collagen (Amide III), and carbonate (v_1_ $CO_{3}^{2-}$) within the implant thread.

BSE-SEM corroborated the histological observations. Within the implant threads as well as around the implant, large amounts of remodeled, highly mineralized, lamellar/osteonal bone were observed (**Figure 6**). Within the implant thread, Raman imaging revealed typical components of mature bone, i.e., apatite (v_1_ $PO_{4}^{3-}$ peak at 960 cm^-1^, v_2_ $PO_{4}^{3-}$ band centered at approximately 432 cm^-1^, v_4_ $PO_{4}^{3-}$ band centered at approximately 580 cm^-1^, and v_1_ $CO_{3}^{2-}$ peak at 1070 cm^-1^) and type-I collagen (amide III band at 1215–1300 cm^-1^).

#### Histological Evaluation of the Soft Tissue Sample

**Figure 7:**
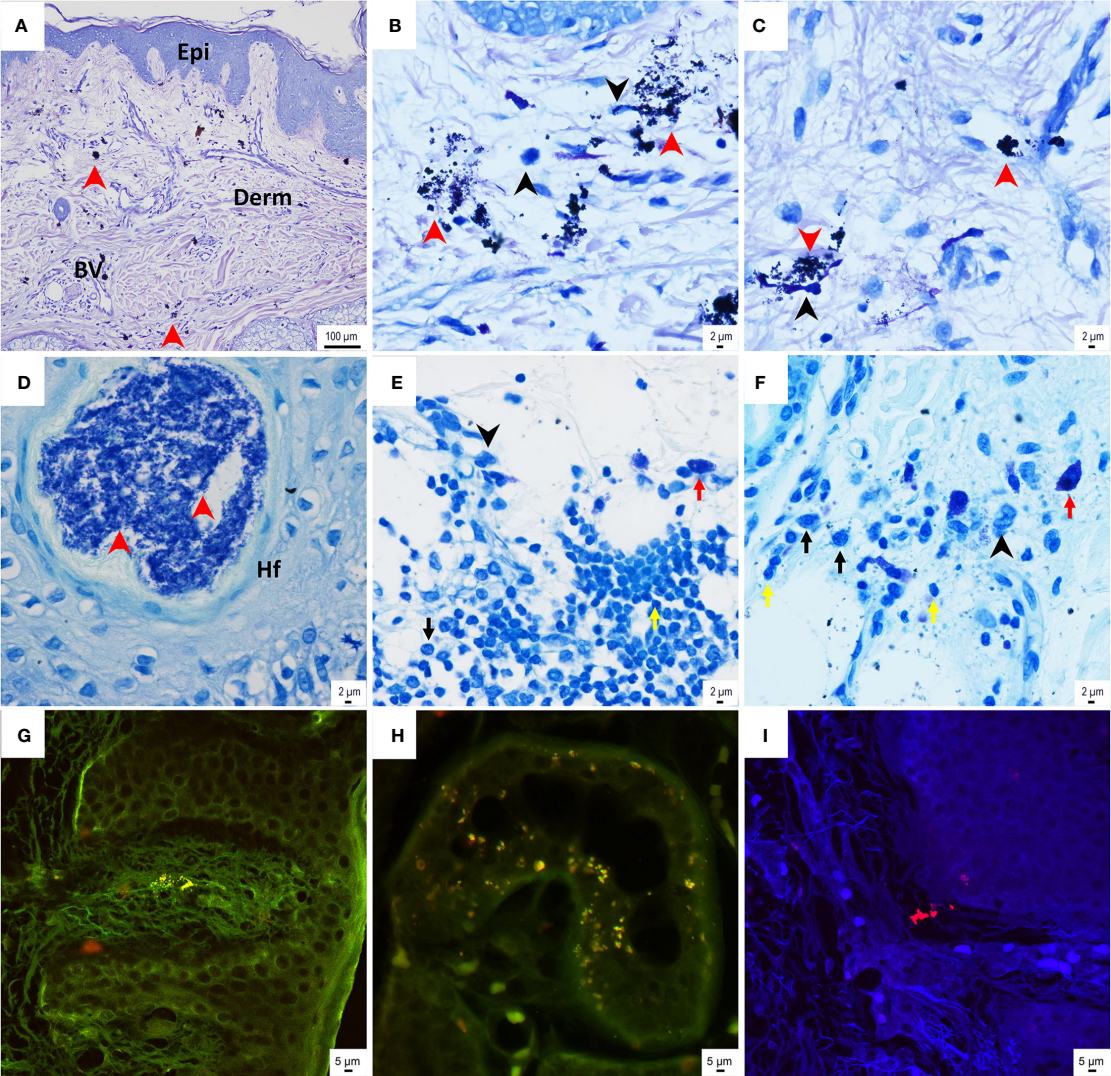
Histological assessment of the skin sample above the BAHS implant. **(A–C)** Giemsa-stained histological sections show the normal appearance of skin structure, consisting of epidermis (Epi) and subepithelial connective tissue dermis (Derm) containing blood vessels (BV). Aggregates of bacterial cells (indicated by the red arrowheads in **A**–**C**), as well as mononuclear/macrophage cells (indicated by the black arrowheads in **B**, **D**), are frequently detected in the connective tissue. Some of the bacterial cells assume intracellular localization **(B)**. The toluidine blue-stained sections also show dense bacterial aggregates in association with hair follicles (red arrowheads in **D**). **(E, F)** Relatively dense inflammatory infiltrates are found in the deepest part of the soft tissue sample, presumably interfacing with the bone where the implant is inserted. The inflammatory infiltrates in **(E**, **F)** consist of mononuclear cells/macrophages, lymphocytes, plasma cells, and mast cells (indicated by black arrowheads and the yellow, black, and red arrows, respectively). **(G–I)** Tissue sections underwent fluorescence *in situ* hybridization (FISH) with a peptide nucleic acid (PNA) probe targeting coagulase-negative staphylococci (CoNS) and *Staphylococcus aureus* in the tissue. CoNS (red cocci of approximately 1 μm) were detected in the tissue, both superficially at the epidermis **(G)** and at deeper layers of the dermis **(H, I)**. *Staphylococcus aureus* (green cocci of approximately 1 μm) was also detected in the tissue **(G, H)**.

The histological assessment of the skin sample, which had grown over the BAHS implant, showed a normal appearance of the epithelial layers of the epidermis and the subepithelial, vascularized, connective tissue dermis (**Figure 7A**). Clusters of darkly stained bacterial cell aggregates were frequently detected in the dermal connective tissue (**Figure 7A-C**). Variable degrees of inflammatory cell infiltrates were detected, consisting predominantly of macrophages and lymphocytes and to a lesser degree of plasma cells and mast cells. However, PMNs were seldom detected (**Figures 7B, C, E, F**). Some of the inflammatory cells assumed interaction with the bacterial cells, indicated by the proximity to the bacterial aggregates as well as the presumed intracellular bacteria (**Figure 7B**). On some occasions, relatively dense bacterial cell aggregates were also found within the skin appendages in association with the hair follicles (**Figure 7D**). A FISH analysis of soft tissue further confirmed the presence of bacteria, such as single cells and clusters, across the epidermis and dermis (**Figure 7G-I**). Both *S. aureus* (green) and CoNS (red) were identified in the soft tissue, as observed by the strong green and red fluorescent cocci (≤1 μm) over the autofluorescent soft tissue in the background.

### Systematic Literature Review

#### Study Selection

The literature search resulted in the identification of 394 studies (**Figure 2**). After removal of duplicates, the titles and abstracts were initially screened for inclusion, resulting in the consideration of 32 publications. After independently reading the full-text (authors MLJ and AP), 6 articles fulfilled the inclusion criteria and were selected for inclusion. The manual search and screening of reference lists resulted in the further addition of 4 articles leading to a total of 10 articles, published between 1987 and 2008, to be included in the review (Jacobsson et al., 1987; Tjellstrom et al., 1988; Holgers et al., 1994a; van der Pouw et al., 1998; Bolind et al., 2000; Granström, 2000; Mylanus et al., 2002; Bolind et al., 2006; Khwaja et al., 2008; Mlynski et al., 2008).

#### Study Characteristics

The articles report the analysis of 54 implants from 47 patients. Of these, 35 were BAHS implants from 35 patients, whereas 19 BAAE implants from 12 patients were evaluated. The time *in situ* varied between 2 weeks and 8 years (**Table 3**). All implants were machined from commercially pure titanium with a diameter of 3.75 mm and a length of 3 or 4 mm manufactured by Cochlear Nordic, Sweden (previously Nobel Pharma and Entific AB, Sweden). Reasons for the 40 elective removals were: non-use of devices or changes in treatment (n=13, 33%), pain (n=11, 28%), adverse skin reactions (n=9, 23%), postmortem (n=4, 10%), tumor excision (n=2, 5%) and mechanical problems (n=1, 3%). In addition, failed implants due to trauma (n=2) or spontaneous loss (n=2) were collected for analysis. Finally, 10 implants were planned for removal from volunteers.

**Table 3:** Characteristics of included studies.

Article	Treatment	No of implants	Implant type and model	No of patients	Age (y)	Gender [M/F]	Time in situ (mo)	Reason for removal	Analytical techniques	Soft tissue samples	Bone/implant samples	BIC & BA[%]	Other notes
Bolind et al. (2000)	BAHS (13) BAAE (1)	14	c.p. titanium 3 and 4 mm length Ø3.75 mm (Nobel Biocare, Sweden)	14p	16-72	M: 7p F: 7p	9-95	Equipment related (n=7) Host response (n=6) Postmortem (n=1)	Histopathology and histomorphometry of resin embedded implant	–	All implants surrounded by lamellar bone with woven bone in the interface undergoing remodeling. Soft tissue, with varying degree of inflammation, present under the flange for most implants	BIC 27.8-87.9 BA 65.3-97.4	
Bolind et al. (2006)	BAAE	5	c.p. titanium 3 and 4 mm length Ø3.75 mm (Entific, Sweden)	2p	50/66	M	4/24	Postmortem (n=3) Tumor excision (n=2)	Histopathology and histomorphometry of resin embedded implant	–	Bone resorption under flange, filled with soft tissue with varying degree of inflammation Patient 1 (4 months in situ): capsular formation partly separating implant from surrounding bone. Patient 2 (loaded for 24 months): Mature and newly formed bone present around the implant	BA 45-53* (for the patient with implant loaded for 24 months) BA 65-88* (for three implants)	Implants placed after irradiation therapy
Granström (2000)	BAHS (3) BAAE (3)	6	c.p. titanium 3 and 4 mm length Ø3.75 mm (Nobel Biocare, Sweden)	5p	9-81	M: 4p F: 1p	24-60	Mechanical problems (n=1) Fistula (n=1) Trauma (n=1) Unable to use the device (n=3)	Histopathology and histomorphometry of resin embedded implant	–	Lamellar bone in contact with the implant. Remodeling in some areas.	BIC 73.6 SD7.2 Range 64.6-83.3 BA Not determined	Longer osseointegration time increased BIC,
Holgers et al. (1994a)	BAAE	1	c.p. titanium 4 mm length Ø3.75 mm, (Nobelpharma, Sweden)	1p	32	M	96	Long period of skin irritation associated with bacterial infection with *S. aureus*	Histopathology of skin biopsy. Bacterial culturing. Histopathology and histomorphometry of resin embedded implant.	Inflammatory reaction with infiltration of PMN, macrophages and lymphocytes. *S. aureus* identified in the skin.	Mature bone with haversian canals in intimate contact with the surface of the implant. No morphological signs of inflammation. There was bone in all threads including the area immediately beneath the flange on one side of the specimen.	BIC 70.1 (SD 17.6) BA 84.3 (SD 17.4)	Abutment removed 14 months prior implant removal. Positive culture of *S. aureus* at implant removal
Jacobsson et al. (1987)	BAHS	1	c.p. titanium Ø3.75 mm (Nobelpharma, Sweden)	1p	–	F	18	Irritation, infection and granulation	Histopathology of skin biopsy. Bacterial culturing. Histopathology and histomorphometry of resin embedded implant.	Inflammatory reaction in the superficial parts of the soft tissue with an accumulation of PMN. *S. aureus* identified in the skin.	Direct contact between bone and implant surface. No signs of inflammation in the bone.	–	Abutment removal after 9 months, reinstalled abutment at 15 months
Khwaja et al. (2008)	BAHS	2	c.p. titanium 4 mm length, Ø3.75 mm, (Cochlear Nordic AB, Sweden)	2p	68/72	M	35/17	Spontaneous loss after six-month history of pain (n=1) Change of treatment (n=1)	Histopathology and transmission electron microscopy of resin embedded bone tissue removed from the implant.	–	Presence of keratinocytes at the bone interfacing the implant. Evidence of bacterial colonies but no biofilm identified (gram-positive).	–	
Mlynski et al. (2008)	BAHS	5	c.p. titanium 4 mm length Ø3.75 mm, (Entific/Cochlear Nordic, Sweden)	5p	3-62 (3/17/50/56/62)	M: 1p F: 4p	Median 12 (0.5/8/12/12/15)	Spontaneous loss (n=1) Pain (n=1) Trauma (n=1) Change of treatment (n=2)	Histopathology of skin and subcutaneous tissue. Histopathology and histomorphometry of resin embedded implant. Scanning electron microscopy.	Chronic inflammation in the skin and subcutaneous tissue in one sample. No foreign body cells were identified.	All implants were well osseointegrated and in contact with a mature lamellar bone (The spontaneously lost implant that showed no osseointegration). No signs of osteomyelitis. SEM revealed processes of mineralized bone appearing to be attached to the implant surface. Small areas of soft tissue/connective tissue under the flange.	39-57 (0 in the spontaneously lost implant)	None of the patients had inflammation or skin reactions prior to removal. Computed tomography of the patient with pain revealed contact of the implant to the dura.
Mylanus et al. (2002)	BAHS (3) BAAE (4)	7	c.p. titanium 4 mm length Ø3.75 mm (Entific, Sweden)	4	17-52	–	BAHS: 5-18 BAAE: 91	Chronic pain. 3 of 4 patients had skin reactions preceded by pain. Immediate onset of pain after implantation for 2 of 4 patients.	Histopathology and histomorphometry of resin embedded implant.	–	Soft tissue under flange in most implants, containing inflammatory cells. Bone resorption was seen in all but one. Bacteria observed in one implant (no evidence provided).	BIC 28-78 BA 48-89	Pain gone or diminished after removal in all but one BAHS patient.
Tjellstrom et al. (1988)	BAHS	10	c.p. titanium 4 mm length Ø3.75 mm, (Nobelpharma, Sweden)	10	27-71	M: 7p F: 3p	3-4	Planned removal. Patients treated with BAHS volunteered to have one extra implant inserted. This was rotated out at the time of the second-stage surgical procedure 3 to 4 months later.	Torque measurements (n=9) Histopathology of one resin embedded implant (n=1)	–	Removal torque: mean 42.7 Ncm, range 26-60 Ncm. Histology revealed areas of bone in direct continuity with the surface of the titanium implant although there was also evidence of some soft-tissue lining (n=1).	–	
van der Pouw et al. (1998)	BAHS	3	c.p. titanium 4 mm length, Ø3.75 mm (Nobel Biocare, Sweden)	3	43/70/71	M: 1p F: 2p	8-31	Pain	Histopathology and histomorphometry of resin embedded implant.	–	The qualitative histologic findings showed no major differences between the three samples. The implants were surrounded and integrated with mature bone except for the implant that had been in situ for 8 months. Here it was surrounded by woven, younger bone undergoing remodeling. A typical feature under the flange of the implant was a soft tissue separation of the implant and bone tissue. Resorption and inflammatory cells were observed in this area. In the area below the apical part of the implant, acellular tissue was observed, seemingly to be bone flakes	BIC 37-61 BA 67-73	No explanation was found for the chronic pain and the relief of it after implant removal.

All implants in these studies were commercially pure (c.p.) titanium, 3 or 4 mm in length and 3.75 mm in diameter (manufactured by Nobelpharma, Nobel Biocare, Entific or Cochlear, Sweden). BAHS=Bone anchored hearing system, BAAE=Bone anchored auricular epithesis, PMN=Polymorphonuclear granulocytes, BIC=Bone to implant contact, BA=Bone area in thread, * for the three best threads.

#### Synthesis of Result From Literature Review

The majority of the implants together with the surrounding bone were embedded in resin, ground sectioned, and evaluated histologically (42 of 53). Thirty-nine specimens were analyzed histomorphometrically with respect to bone-to-implant contact (BIC) and bone area in threads (BA). The BIC ranged between 27.8-87.9%, with the lower values obtained for the implants with short *in situ* periods, whereas the BA ranged between 48.0-97.4%. All implants, except the two spontaneously lost and one explanted (4 months *in situ*), were surrounded by and integrated with the bones of varying maturity, depending on time *in situ*. A common finding was the absence of bone tissue below the proximal flange down to approximately the first thread of the implant. This area was instead occupied by soft tissue containing a varying amount of inflammatory cells, such as polymorphonuclear granulocytes (PMNs), indicating sub-acute to chronic inflammation. The adjacent bone showed signs of bone resorption in some samples. Two studies reported that the area below the apical part of the implant contained acellular tissues, which were seemingly bone flakes originating from the creation of the osteotomy (van der Pouw et al., 1998; Mylanus et al., 2002).

One study analyzed resin-embedded bone tissue removed from the implant using histopathology and transmission electron microscopy, reporting the presence of keratinocytes in the bone adjacent to the implant surface (Khwaja et al., 2008). Evidence of gram-positive bacterial cells was also detected; however, no biofilm was identified. Three implants, one spontaneous loss and two from elective removal were analyzed using scanning electron microscopy. The ultrastructural interaction between the surrounding bone as well as osteocyte processes attaching to the implant surface for the two electively removed implants was demonstrated; however, none of these features were present in the extruded implant (Mlynski et al., 2008). Nine of the ten implants inserted in the volunteers were removed using a torque gauge instrument after 3-4 months, with a mean removal torque of 42.7 Ncm (range 26 to 60 Ncm) (Tjellstrom et al., 1988).

None of the studies included the abutment with its surrounding soft tissue in the resin embedded ground sections. However, in one case where the implant was removed due to adverse skin reactions, several skin biopsies and peri-abutment bacterial cultures were obtained at different time points. At implant retrieval, an inflammatory reaction was detected in the superficial parts of the skin tissue with accumulations of PMNs and granulation tissue, together with the presence of *Staphylococcus aureus* (Jacobsson et al., 1987). The deeper parts of the soft tissues and the bone tissue were, however, free of inflammatory reactions. In one study, skin and subcutaneous tissue were collected from five explants (Mlynski et al., 2008). Lymphocytes and granular infiltrates, indicating chronic inflammation, were revealed in only one of the cases. One implant was removed after 8 years following a long period of skin irritation, which on two occasions was associated with bacterial infection with *S. aureus*, despite local treatment with steroid/antibiotic ointment and skin grafts (Holgers et al., 1994a). The abutment was removed, and 14 months later the implant was removed. A morphological analysis of the soft tissue above the implant demonstrated pronounced infiltration of PMNs but also macrophages and lymphocytes. Despite the inflammatory reaction in the soft tissue, a surprisingly high degree of bone-implant contact was revealed.

## Discussion

In recent decades, BAHS has become an established method of audiological rehabilitation with high implant survival rates (Badran et al., 2009; Dun et al., 2012; Calon et al., 2018b; Lagerkvist et al., 2020). Osseointegration is a proven concept in BAHS, dental rehabilitation, and prosthetic treatment of patients with an amputated femur bone. The present study entails an elaborate case report of a patient with a clinically stable and osseointegrated implant reporting pain that persisted after abutment removal. This case patient was part of a larger controlled study evaluating the outcome after MIPS surgery compared with linear incision (Calon et al., 2018a; Strijbos et al., 2021). This gave the opportunity to apply a panel of modern analytical techniques on a specific, clinically used devices in addition to the available follow-up data. Here, the first case report showing chronic infection of BAHS as a possible explanation for chronic pain is provided. The present systematic literature review showed that few BAHS and BAAE implants have been retrieved and analyzed along with a presentation of detailed clinical and analytical data.

Pain related to BAHS implantation is an important complication that might, in addition to the obvious morbidity for the individual patient, result in reduced usage of the sound processor. Moreover, the pain might hide other underlying causes, such as infection. However, the etiology is not fully understood (Siau et al., 2012). As with numbness, pain is subjective and patient-dependent. The degree of pain is typically reported as a dichotomous outcome (present; yes or no) or in a few reports on a visual analog scale. Pain related to BAHS can be categorized as postsurgery and chronic or recurrent. Limited postoperative pain is reported for 2-9% of visits up to one year (Hogsbro et al., 2015; Mowinckel et al., 2016; Caruso et al., 2017; Johansson et al., 2017). Hogsbro *et al.* demonstrated that when using a tissue preservation technique, no pain was observed after one year, compared to 7% when using a tissue reduction (dermatome) technique (Hogsbro et al., 2015). The incidence of chronic or recurrent pain for BAHS patients is reported to be between 1.2-4.2% (van der Pouw et al., 1998; Badran et al., 2009; Siau et al., 2012; Caspers et al., 2019). Similarly, delayed and persistent pain around a cochlear implant (CI) receiver/stimulator occurs in 2.8% of patients (Shapira et al., 2015). Even though the reason for the pain could not be determined for these CI patients, the authors speculated that biofilm formation on the implant or loss of hermicity could be explanations.

### Electively Removed Implants

Previous retrospective studies have shown that BAHS implants are electively removed for reasons including idiopathic pain, adverse skin reactions, patient preference, and non-use of hearing devices at a rate between 1.6 and 7.2% (Mlynski et al., 2008; Badran et al., 2009; Dun et al., 2012; Siau et al., 2012; Calon et al., 2018b). Dun et al. reported elective removal in 1.6% (16 of 1086 implants), and of these, 37.5% (6 of 16) were removed due to chronic pain. Siau et al. found a 4.5% elective removal rate (27 of 299). The main reasons for removal were chronic pain without clinical manifestation of infection or inflammation in 12 patients (44%, or 2% of the total cohort) and chronic infection in 7 cases (26%, or 1.3% of the total cohort) (Siau et al., 2012). Badran et al. reported a total incidence of elective removal of 7.2% (12 of 165 patients). Seven (4.2%) patients experienced chronic pain, leading to removal for four of them (2.4%) (Badran et al., 2009). Hence, in addition to non-usage or patient preferences, chronic pain and chronic soft tissue reactions seem to be the main drivers for elective removal, which is also reflected in the systematic literature review where 28% of the analyzed explants were removed due to pain. The review also demonstrated that the main reason for removal was lack of benefit or a planned change of treatment. Change of treatment, requiring removal of the BAHS implant could be expected to be increased in the future due to the emergence of additional treatment possibilities such as active and passive bone-conduction implants for this patient group.

### Osseointegration

In the present case study, histology, BSE-SEM and micro-CT all provided evidence that there was a high degree of osseointegration. The systematic literature review revealed that the estimated bone-to-implant contact varied between 28-88% for stable implants with increased values over time (van der Pouw et al., 1998; Bolind et al., 2000; Granström, 2000; Mylanus et al., 2002; Bolind et al., 2006; Mlynski et al., 2008). This finding is in line with the observed 57.7% BIC in this case study.

In addition, the literature review revealed a common finding that, under the flange, an area with less bone contact was observed (Tjellstrom et al., 1988; van der Pouw et al., 1998; Bolind et al., 2000; Granström, 2000; Mylanus et al., 2002; Bolind et al., 2006; Mlynski et al., 2008). In contrast, the visual assessment indicates that the retrieved implant presented in this case report has a higher degree of bone-to-implant contact under the flange. This difference might be attributed to implant design. In the studies included in the literature review, all the extracted implants had a diameter of 3.75 mm and a flange of 5-5.5 mm in diameter. The present case study, however, represents the first retrieved Ponto Wide Implant that has been investigated, which is a newer generation of wide diameter implant (Ø4.5 mm, 5 mm flange). Currently, implants for BAHS and BAAE applications exclusively use these implants with a wider diameter. These are either used as machined (turned), with surface modifications, such as laser ablation (Shah et al., 2016) or blasting (den Besten et al., 2016) or design features such as microthreads intended to distribute axial loads and generate stresses to the surrounding bone more evenly (Hansson, 1999) and thereby reducing the risk of bone resorption. A long-term survival rate of 93.9-97.7% in adult populations has been reported (den Besten et al., 2016; Lagerkvist et al., 2020), which is higher than that of smaller implants (92.7%) (Dun et al., 2012). Possibly, the higher degree of bone under the flange may be attributable to an improved implant design. However, this should be evaluated systematically in future studies.

### Bacterial Colonization

Multiple microbiological sampling and different analytical techniques were applied to the present case. At baseline and at the 12-week follow-up, mainly normal skin bacteria were observed. However, during episodes of inflammation, the bacterial diversity increased in the peri-abutment skin, including other bacteria, such as *Escherichia coli*, *Haemophilus parainfluenzae*, and *Enterococcus faecalis*. During follow-up, pain complaints were present, although the bacterial species altered over time. Previously, in dental implants, pain was associated with IL-6 and IL-8 expressions (Sayardoust et al., 2017). Although we could not confirm these results for BAHS (Calon et al., 2018c), a similar mechanism might be attributable to pain complaints in this patient, irrespective of which specific bacterial species were present. Additionally, in this case, it cannot be excluded that the immune system might have been impaired due to her diabetes (Geerlings and Hoepelman, 1999; Muller et al., 2005). Although this risk is decreased with well-controlled diabetes, an increased prevalence of implant loss and adverse soft tissue reactions in BAHS patients with type 2 diabetes mellitus has been reported (Horstink et al., 2012; Kellermeyer et al., 2020).

Only later, at the time of abutment removal, *Staphylococcus aureus* was observed in high amounts. Strikingly, the bacterial profile on the implant strongly resembled the profile found on the abutment prior to implant removal, indicating that the infection was maintained on the implant level over 10 months of soft tissue healing. In addition, the soft tissue contained several areas of persistent staphylococci, as confirmed by FISH. Moreover, bacteria-like cell structures were observed in the peri-implant bone, suggesting bacterial colonization in the bone. The subject complained of episodes of pain with tenderness of the soft tissue around the implant, while macroscopically the skin and bone showed no clear signs of infection (**Figures 1B, C**). The presence of bacteria and subclinical infection are plausible explanations for these complaints. However, confirmation of these results is warranted (i.e., by culturing the causative pathogen).

Previous studies have shown the presence of several different species of bacteria in relation to BAHS as well as bacterial colonization of implants and abutments (Holgers et al., 1995b; Monksfield et al., 2011; Trobos et al., 2018). Cultures from both noninflamed and inflamed BAHS sites have identified CoNS (especially *S. epidermidis*) and *S. aureus* (Holgers et al., 1992; Holgers et al., 1994a; Holgers and Ljungh, 1999; Trobos et al., 2018). The present study, using the detection of bacterial DNA by the IS-pro technique, confirms their presence and possible role in adverse reactions associated with BAHSs.

In the present case study, the samples analyzed with IS-pro used bacterial swabs obtained from the cover screw and the top of the implant. An important observation was that bacteria were also detected in the peri-abutment soft tissue with indications of their presence in the peri-implant bone. If we assume that bacterial colonization is indeed present in the peri-abutment soft tissue with indications of their presence in the peri-implant bone, it is striking that the implant itself was well integrated. In addition, ISQ, a suggested surrogate for implant stability (Meredith et al., 1996), remained stable over time. While the immune responses around implants are assumed to be impaired (Broekhuizen et al., 2008; Busscher et al., 2012), an equilibrium might be present, preventing complete bacterial colonization of the implant while bacteria persist intracellularly. Shifts in this equilibrium or in bacterial flora composition and mechanical load might also be explanations for the episodes of tenderness described by the patient.

Apart from visit 4 (after 24 days), visit 6 (after 6 months) and visit 12 (after 14 months), the highest level of pain was reported by the patient. When evaluating the bacterial and gene expression profiles at these time points, the following observations could be made. First, two distinct bacterial flora compositions were detected, with a constant prevalence and increasing abundance of *S. epidermidis* over time. The species *S. epidermidis* is considered to be a permanent colonizer of human skin and an important etiological agent of implant-associated infections, particularly those with a more chronic course (Otto, 2009). While not producing aggressive virulence factors, it is highly possible that the implant becomes contaminated during insertion, eventually becoming an opportunistic pathogen (Uckay et al., 2009). The IS-Pro results showed that the presence of *S. epidermidis* in the skin-abutment-implant compartments was ubiquitous, and its relative abundance increased over time, which could have driven, at least partly, the chronic pain experienced by the patient. Second, *S. aureus* was detected toward the end of the implant *in situ* period (visit 12-14 = after 14-25 months), corresponding to clinical signs of irritation, pain, removal of abutment and implant explantation. Contrary to *S. epidermidis*, *S. aureus* commonly causes acute and virulent implant-associated infections (Otto, 2009). The presence and high abundance of *S. aureus* at this stage could have been linked to the documented pain and irritation, leading to the removal of the abutment and implant.

Recent findings have shown that sensory neurons have a microbial detection mechanism leading to neuronal activation as well as pain and itching (Baral et al., 2016). *S. aureus*, detected in the present case, might induce pain *via* action potential generation in nociceptor neurons (Chiu, 2018). Similar to the immune system, one mode of sensory neuron pathogen recognition is *via* receptors, such as toll-like receptors (TLRs). Furthermore, neuron-immune cell crosstalk modulates the cellular response and activation. Neurons are activated by immune cell-derived cytokines (e.g., IL-1b and TNF-α), chemicals (e.g., histamine and bradykinin), and lipid mediators (e.g., prostaglandins), thus leading to pain and itching. Neurons, in turn, release mediators that modulate immune cell function. Hence, although pain and itch are symptoms of inflammation, neurons also play a role in regulating the immune response and host defense. Interestingly, while typical antimicrobial host defense mechanisms, such as immune cell recruitment and cytokine production, are initiated minutes to hours after infection, neurons are able to respond within milliseconds of encountering pathogens (Baral et al., 2016). Although speculative and not possible to elucidate for this case, persistent microbial stimulation might provide one explanation for the pain complaints, despite any macroscopic evidence of infection.

### Tissue Response

In the present systematic literature review, only two studies evaluated the soft tissue around the abutment for two cases revealing inflammation. In addition to these two studies, Holgers and coworkers analyzed the soft tissue adjacent to percutaneous implants in the temporal bone using histological and immunohistochemical techniques in a series of papers (Holgers et al., 1989; Holgers et al., 1994a; Holgers et al., 1994b; Holgers et al., 1995a; Holgers et al., 1995b). In summary, these studies showed a high number of inflammatory and immune cells in association with the abutment even in clinically non-irritated peri-abutment soft tissue. Clinically observed irritated soft tissue demonstrated elevated number of peri-abutment inflammatory and immune cells, indicating a peri-implant cellular barrier which is alerted upon exogenous stimuli.

A recent prospective clinical study used qPCR to demonstrate an upregulation of genes related to inflammation (IL-1b, IL-8) and tissue remodeling (COL1a1, MMP-9, and TIMP-1) within the soft tissue close to the abutment 12 weeks after implantation in BAHS, compared with the baseline. Furthermore, the expression of IL-1β, IL-17 and TNF-a was higher in patients experiencing adverse soft tissue reactions (inflammation) than in noninflamed patients (Calon et al., 2018c). An observational study analyzing fluid exudate at noninflamed and inflamed BAHSs for the detection and quantitation of secreted proteins revealed significantly higher concentrations of the pro-inflammatory cytokines IL-1b, TNF-a, and TIMP1 in inflamed BAHSs than in noninflamed BAHSs (Grant et al., 2010). In agreement with the latter findings, the expression of genes denoting inflammation (IL-1ß, IL-6, TNF-α, MIP-1α) was upregulated compared with that in a noninflamed state.

### Patient Related Factors

In experimental models, diabetes has been found to be associated with impaired osseointegration and wound healing (Gerritsen et al., 2000). Similarly, in BAHS applications, diabetes has been found to be related to a higher degree of implant loss whereas Body Mass Index (BMI) has been associated with soft tissue reactions (Horstink et al., 2012; Rebol, 2015; Candreia et al., 2016). However, other studies have found no relationship between BMI and diabetes with soft tissue reactions or implant loss (den Besten et al., 2015). The patient in the case reported here was installed with a 12 mm abutment. In the randomized controlled trial, in which this patient was enrolled, the 3 months follow-up data included a statistical model for implant stability in terms of ISQ, demonstrating that implants with a 12 mm abutment had a mean ISQ value of 51 directly post-implantation (Calon et al., 2018a). This is equal to the primary ISQ value for the individual patient reported here. Hence, the stability values for the case patient and the further rise during follow-up were consistent with normal values in the trial. Although there were no indications of an unstable implant, the presence of diabetes and a relative thick skin could be relevant factors associated with a chronic infection.

### Clinical Considerations

Chronic pain related to BAHS is a known problem that can hinder overall satisfaction, increase morbidity and medical consultation, and can even lead to implant removal (Badran et al., 2009; Siau et al., 2012; Calon et al., 2018b). Titanium allergy, dura contact, and bacterial presence have been postulated as possible explanations for these complaints (van der Pouw et al., 1998; Mylanus et al., 2002; Addison et al., 2012). In clinical practice, pain complaints without specific macroscopic signs of inflammation are sometimes pragmatically treated with local or systemic antibiotics, but no clear treatment strategy is defined (Kruyt et al., 2017). A recent retrospective chart analysis of BAHS patients with idiopathic pain showed that in approximately 40% of patients, the pain was resolved with oral antibiotic combination treatment (Caspers et al., 2019). A newly designed scale for soft tissue assessment in bone conduction implants specifically mentions pain as a possible reason for antibiotic treatment (Kruyt et al., 2017). In reviewing the literature on causes and management strategies for delayed pain post cochlear implantation (CI) surgery, without clinical evidence of inflammation or infection, it was found that oral therapy (analgesia, nonsteroidal anti-inflammatories, antibiotics) and local treatments (topical, injections) resolved pain in 41% and 63% of patients, respectively. A total of 33% of patients in this review required explantation of the device, with complete resolution of pain in all of these patients (Sethi et al., 2020). Additionally, for the patient described in the present case report, the pain diminished at the first follow-up visit after implant removal. Our findings support the recommendation to treat idiopathic pain with oral antibiotic combination treatment. If unresponsive to conservative treatment, removal of the implant might be necessary (Siau et al., 2012).

### Diagnostics

Although treatment of pain may involve systemic or local antibiotic agents, it is matter of urgency to arrive at a diagnosis of infection prior to treatment. A dilemma presents itself considering the best method for the diagnosis of chronic (peri-)implant infection without the removal of the device itself. The BAHS is a percutaneous device resulting in permanent bacterial colonization through the skin breach. Both conventional bacterial cultures and molecular techniques (IS-pro, whole-genome sequencing or PCR) seem suitable techniques for the detection of bacteria on BAHSs (Trobos et al., 2018; Calon et al., 2019). Knowledge of which bacterial species are present at the implant level in patients without pain is lacking; however, there is recent mounting evidence of a link between the presence of *S. aureus* and *S. epidermidis* in cases of inflammation (Calon et al., 2019). Due to the titanium composition of the BAHS, imaging by means of MRI or CT is unlikely to yield reliable outcomes with respect to chronic bone inflammation. As judged by the findings in the present study, further studies are needed to determine if sampling from any of the two most easily accessible peri-implant compartments (abutment, peri-implant crevice) is sufficient, or if small biopsies of the skin are mandatory for culture or molecular analysis to achieve an infection diagnosis. Histological evaluation of soft tissue biopsies taken from the peri-abutment might yield some evidence of soft tissue invasion with bacteria and inflammatory cells. Recently, the use of paper points, a less invasive sampling technique, has been shown to be feasible for evaluating peri abutment bacteria (Trobos et al., 2018). In addition, the composition of inflammatory cells and cytokine expression profiles might provide deepened insight into the infection diagnosis or the inflammation of the peri-abutment skin. Although one should consider the risks for complications of a biopsy, multimodal evaluation of soft tissue combined with a peri-implant bone biopsy might yield the most conclusive evidence for chronic infections of the peri-implant bone.

Diagnosis is indeed an essential step when addressing adverse reactions and clinical events may occur in different compartments. Therefore, a combination of different techniques may be needed for an accurate diagnosis: clinical signs and symptoms, laboratory signs of infection, microbiology, histology, and imaging. However, from a clinical perspective some of the explorative techniques, which was applied in the present case, may not be readily available. Hence, at this stage it is rather premature to arrive at definite recommendations in cases of subclinical infections in combination with idiopathic pain. This report presents an in-depth analysis of a single patient and both the clinical and research community are in need of more information. Although evidence for the best approach for idiopathic pain in BAHS and BAAE is lacking, we propose the following stepwise approach in patients with pain complaints: (1) Local treatment with hygienic advice, (2) Sampling (swabs or paper-points from abutment-skin interface), diagnostic microbiological cultures and susceptibility testing of causative pathogen, and initiation of oral antibiotics, (3) Broad spectrum oral antibiotics, (4) Abutment removal and (5) Implant removal. In case of removal of abutment and implant, these should preferably be retrieved, preserved and analyzed.

### Future Studies

To gain further understanding of osseointegration, a detailed evaluation of clinically retrieved implants is needed. Elective implant removal is quite rare. However, when removal is requested, it is usually associated with a relatively high degree of morbidity. Although uncommon, chronic pain is one of the most common reasons for implant removal and deserves increased attention. Biofilms have been shown to be present on BAHSs (Monksfield et al., 2011; van Hoof et al., 2015). Implant-associated infections can pose a challenge in clinical practice, especially in cases of biofilm formation leading to chronic infections and increased resistance to antibiotic treatment (Francolini and Donelli, 2010). Device removal may be necessary in these cases for the complete eradication of the infection. As presented in this article, several techniques can be employed in cases of implant removal to increase our knowledge of osseointegration, chronic pain, and implant-associated infection and inflammation. Larger case series using various techniques to determine osseointegration and sensitive procedures for bacterial detection are needed to confirm our results.

### Retrieval, Processing, Preparation, and Analysis of Clinical Implants

**Figure 8:**
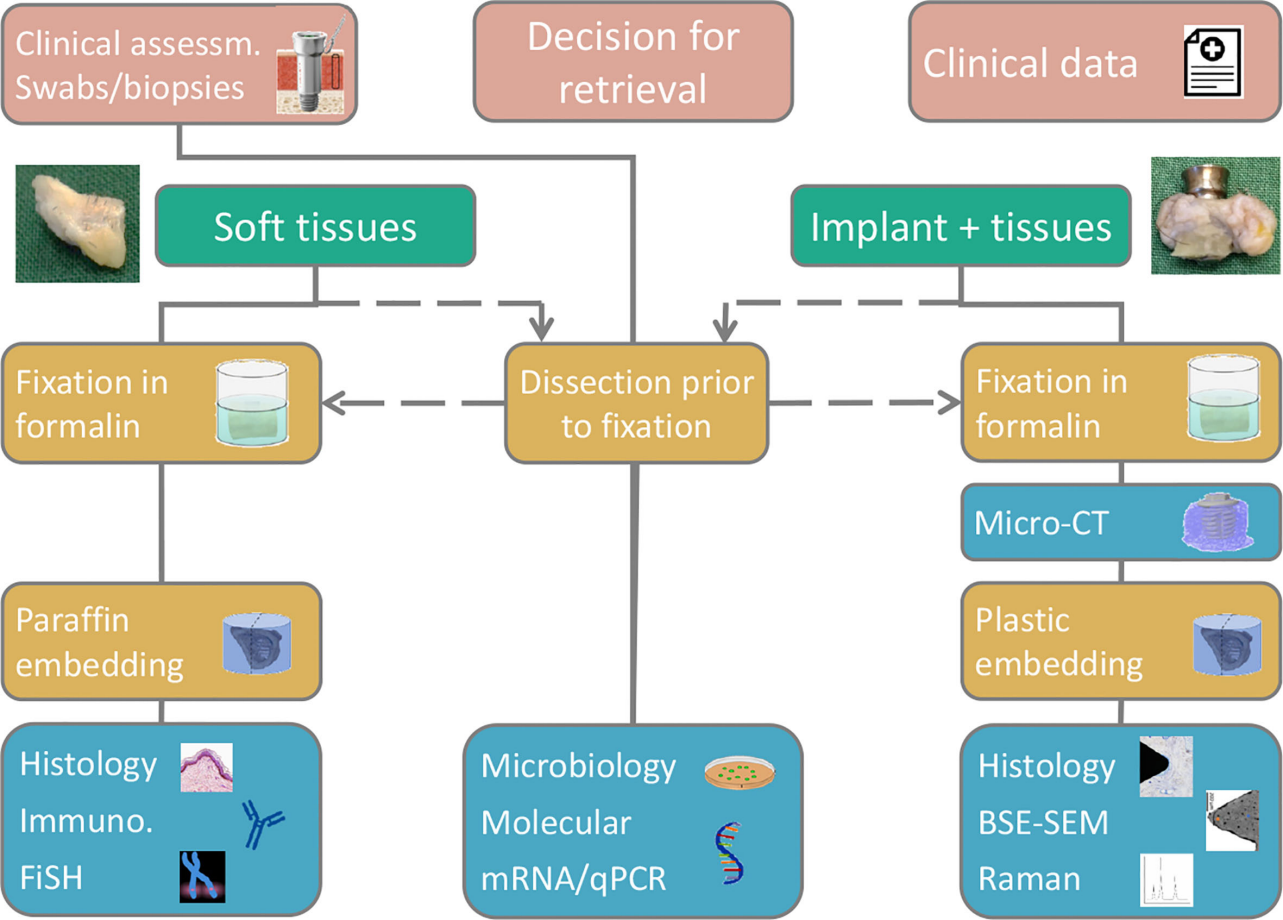
A suggested flow chart for retrieval analyses, enabling a multimodal and multiscale approach, adapted for the Implant Retrieval Network. Depending on sample type retrieved (soft tissue, hard tissue, implant, abutment) various analytical methods are available, however, via different sample preservation and preparation routes.

The importance of a detailed understanding of how a given implant design performs in humans cannot be overstated. We currently lack insight into the biological processes at and around bone-conducting implants because most of the knowledge is based on rather subjective clinical observations. Therefore, the mechanisms leading to success as well as complications, such as inflammation, infection, and failure, are poorly understood. Implant retrievals may allow for a detailed analysis of an implant and its surrounding tissue, as demonstrated for the present case, particularly if a multimodal and multiscale analytical approach is adopted. To advance this, we created a collaborative implant retrieval network with leading European clinics and universities to collect and analyze retrieved implants. The aim of this initiative is to provide a detailed characterization of tissue compartments using multiple analytical techniques and to correlate the clinical data with the underpinning microbiological, molecular, and morphological fingerprints at the tissue interface (**Figure 8**). Therefore, we aim to increase the understanding of the mechanisms underlying the success and failure of different BAHS treatments.

The etiology of chronic pain is indeed a clinically challenging problem and increased understanding of this issue is essential. In cases when implant removal is deemed necessary, we suggest adhering to a systemic approach (**Figure 8**). By following this approach, increased knowledge on adverse events is gained and may provide increased evidence-based treatments in the future.

## Conclusion

In this report we provide a detailed analysis to understand the possible mechanisms in the challenging case of chronic pain after BAHS. Extensive analyses show clear evidence of osseointegration of the retrieved Ponto Wide Implant using complementary techniques. Here, we provide evidence suggesting that chronic pain related to BAHSs can result from a chronic bacterial infection with observed intracellular bacteria, even when no macroscopic signs of an infection are present. To further improve understanding on osseointegration and adverse events in BAHS, we introduce a flow chart for retrieval analyses, linking clinical findings with the status of the tissues surrounding the implant. A lack of literature is revealed by the very few cases found in the systematic review part, indicating the need for further case studies/series.

## Data Availability Statement

The raw data supporting the conclusions of this article will be made available by the authors, without undue reservation.

## Ethics Statement

The studies involving human participants were reviewed and approved by Medical ethics committee, METC, azM/UM, Maastricht, The Netherlands). The patients/participants provided their written informed consent to participate in this study. Written informed consent was obtained from the individual(s) for the publication of any potentially identifiable images or data included in this article.

## Author Contributions

MJ and TC executed the study, were involved in the different experiments and their analyses, and wrote the paper with input from all authors. MT were involved in performing and analyzing IS-pro. OO and PT were involved in analyzing the gene expression data. MJ, PT, and OO performed the histological and histomorphometrical analyses. AP performed the micro-CT experiment. FS and AP performed the Raman spectroscopy. MT performed the FISH experiment. RS performed the surgeries. MJ and AP performed the literature review. RS, PT and AP supervised the study. All authors contributed to the article and approved the submitted version.

## Funding

This study was sponsored by Oticon Medical AB, Sweden and supported by the Swedish Research Council (2018-02891, 2020-04715), the Swedish state under the agreement between the Swedish government and the county councils, the ALF agreement (ALFGBG-725641), the Eivind o Elsa K:son Sylvan Foundation, the Hjalmar Svensson Foundation, Adlerbertska Forskningsstiftelsen (GU 2019/86, 13/09/19), the IngaBritt and Arne Lundberg Foundation, CARe – Centre for Antibiotic Resistance Research at University of Gothenburg and the Area of Advance Materials of Chalmers and GU Biomaterials within the Strategic Research Area initiative launched by the Swedish Government. The patient was enrolled in a sponsor-initiated study by Oticon Medical AB, Sweden (C50/NCT02438618). The sponsor was involved in the study design and analysis. All procedures were in accordance with ISO 14155:2011 and the Declaration of Helsinki. The investigators had access to all data related to the case.

## Conflict of Interest

MJ was employed by company Oticon Medical AB (Askim, Sweden).

The remaining authors declare that the research was conducted in the absence of any commercial or financial relationships that could be construed as a potential conflict of interest.

## References

[B1] Addison O. Davenport A. J. Newport R. J. Kalra S. Monir M. Mosselmans J. F.. (2012). Do ‘passive’ medical titanium surfaces deteriorate in service in the absence of wear? J. R Soc. Interface. 9 (76), 3161–3164. 10.1098/rsif.2012.0438 22832360 PMC3479928

[B2] Badran K. Arya A. K. Bunstone D. Mackinnon N. (2009). Long-term complications of bone-anchored hearing aids: a 14-year experience. J. Laryngol. Otol. 123 (2), 170–176. 10.1017/S0022215108002521 18492306

[B3] Baral P. Mills K. Pinho-Ribeiro F. A. Chiu I. M. (2016). Pain and Itch: Beneficial or Harmful to Antimicrobial Defense? Cell Host Microbe 19 (6), 755–759. 10.1016/j.chom.2016.05.010 27281567

[B4] Bolind P. Acton C. Albrektsson T. Bonding P. Granström G. Johansson C.. (2000). Histologic evaluation of retrieved craniofacial implants. Otolaryngol. Head Neck Surg. 123 (1), 140–146. 10.1067/mhn.2000.104667 10889497

[B5] Bolind P. Johansson C. B. Johansson P. Granström G. Albrektsson T. (2006). Retrieved Implants from Irradiated Sites in Humans: A Histologic/Histomorphometric Investigation of Oral and Craniofacial Implants. Clin. Implant. Dent. Relat. Res. 8 (3), 142–150. 10.1111/j.1708-8208.2006.00010.x 16919022

[B6] Broekhuizen C. A. de Boer L. Schipper K. Jones C. D. Quadir S. Vandenbroucke-Grauls C. M.. (2008). Staphylococcus epidermidis is cleared from biomaterial implants but persists in peri-implant tissue in mice despite rifampicin/vancomycin treatment. J. BioMed. Mater. Res. A. 85 (2), 498–505. 10.1002/jbm.a.31528 17729261

[B7] Budding A. E. Grasman M. E. Lin F. Bogaards J. A. Soeltan-Kaersenhout D. J. Vandenbroucke-Grauls C. M.. (2010). IS-pro: high-throughput molecular fingerprinting of the intestinal microbiota. FASEB J. 24 (11), 4556–4564. 10.1096/fj.10-156190 20643909

[B8] Budding A. E. Hoogewerf M. Vandenbroucke-Grauls C. M. Savelkoul P. H. (2016). Automated Broad-Range Molecular Detection of Bacteria in Clinical Samples. J. Clin. Microbiol. 54 (4), 934–943. 10.1128/JCM.02886-15 26763956 PMC4809945

[B9] Busscher H. J. van der Mei H. C. Subbiahdoss G. Jutte P. C. van den Dungen J. J. Zaat S. A.. (2012). Biomaterial-associated infection: locating the finish line in the race for the surface. Sci. Transl. Med. 4 (153), 153rv110. 10.1126/scitranslmed.3004528 23019658

[B10] Calon T. G. van Hoof M. van den Berge H. de Bruijn A. J. van Tongeren J. Hof J. R.. (2016). Minimally Invasive Ponto Surgery compared to the linear incision technique without soft tissue reduction for bone conduction hearing implants: study protocol for a randomized controlled trial. Trials 17 (1), 540. 10.1186/s13063-016-1662-0 27829464 PMC5103483

[B11] Calon T. G. A. Johansson M. L. de Bruijn A. J. G. van den Berge H. Wagenaar M. Eichhorn E.. (2018a). Minimally Invasive Ponto Surgery Versus the Linear Incision Technique With Soft Tissue Preservation for Bone Conduction Hearing Implants: A Multicenter Randomized Controlled Trial. Otol. Neurotol. 39 (7), 882–893. 10.1097/MAO.0000000000001852 29995008 PMC6075882

[B12] Calon T. G. A. van Tongeren J. Heuft A. M. E. Brunings J. W. Bollen D. Hof J. R.. (2018b). Percutaneous bone-anchored hearing system implant survival after 550 primary implant surgeries. Clin. Otolaryngol. 43 (2), 735–739. 10.1111/coa.13036 29168329

[B13] Calon T. G. A. van Tongeren J. Omar O. Johansson M. L. Stokroos R. J. (2018c). Cytokine expression profile in the bone-anchored hearing system: 12-week results from a prospective randomized, controlled study. Clin. Implant. Dent. Relat. Res. 20 (4), 606–616. 10.1111/cid.12615 29701288 PMC6099213

[B14] Calon T. G. A. Trobos M. Johansson M. L. van Tongeren J. van der Lugt-Degen M. Janssen A. M. L.. (2019). Microbiome on the Bone-Anchored Hearing System: A Prospective Study. Front. Microbiol. 10, 799. 10.3389/fmicb.2019.00799 31105654 PMC6498861

[B15] Candreia C. Birrer R. Fistarol S. Kompis M. Caversaccio M. Arnold A.. (2016). Predisposing factors for adverse skin reactions with percutaneous bone anchored hearing devices implanted with skin reduction techniques. Eur. Arch. Otorhinolaryngol. 273 (12), 4185–4192. 10.1007/s00405-016-4106-2 27250841

[B16] Caruso A. Giannuzzi A. L. Sozzi V. Sanna M. (2017). Bone anchored hearing implants without skin thinning: the Gruppo Otologico surgical and audiological experience. Eur. Arch. Otorhinolaryngol. 274 (2), 695–700. 10.1007/s00405-016-4305-x 27637751

[B17] Caspers C. J. I. Nelissen R. C. Verhamme L. M. Meijer F. J. A. Mylanus E. A. M. Hol M. K. S. (2019). Clinical Presentation, Management, and Outcomes of Idiopathic Pain in Percutaneous Bone-anchored Hearing Implants. Otol. Neurotol. 40 (10), 1292–1298. 10.1097/mao.0000000000002382 31725591

[B18] Chiu I. M. (2018). Infection, Pain, and Itch. Neurosci. Bull. 34 (1), 109–119. 10.1007/s12264-017-0098-1 28144843 PMC5799131

[B19] Cranendonk D. R. Hugenholtz F. Prins J. M. Savelkoul P. H. M. Budding A. E. Wiersinga W. J. (2019). The Skin Microbiota in Patients Hospitalized for Cellulitis and Association With Outcome. Clin. Infect. Dis. 68 (8), 1292–1299. 10.1093/cid/ciy709 30321312

[B20] de Meij T. G. Budding A. E. de Groot E. F. Jansen F. M. Frank Kneepkens C. M. Benninga M. A.. (2016). Composition and stability of intestinal microbiota of healthy children within a Dutch population. FASEB J. 30 (4), 1512–1522. 10.1096/fj.15-278622 26655704

[B21] den Besten C. A. Nelissen R. C. Peer P. G. Faber H. T. Dun C. A. de Wolf M. J.. (2015). A Retrospective Cohort Study on the Influence of Comorbidity on Soft Tissue Reactions, Revision Surgery, and Implant Loss in Bone-anchored Hearing Implants. Otol. Neurotol. 36 (5), 812–818. 10.1097/MAO.0000000000000745 25811351

[B22] den Besten C. A. Stalfors J. Wigren S. Blechert J. I. Flynn M. Eeg-Olofsson M.. (2016). Stability, Survival, and Tolerability of an Auditory Osseointegrated Implant for Bone Conduction Hearing: Long-Term Follow-Up of a Randomized Controlled Trial. Otol. Neurotol. 37 (8), 1077–1083. 10.1097/MAO.0000000000001111 27482783 PMC4982756

[B23] Donath K. Breuner G. (1982). A method for the study of undecalcified bones and teeth with attached soft tissues. The Sage-Schliff (sawing and grinding) technique. J. Oral. Pathol. 11 (4), 318–326. 10.1111/j.1600-0714.1982.tb00172.x 6809919

[B24] Dun C. A. Faber H. T. de Wolf M. J. Mylanus E. A. Cremers C. W. Hol M. K. (2012). Assessment of more than 1,000 implanted percutaneous bone conduction devices: skin reactions and implant survival. Otol. Neurotol. 33 (2), 192–198. 10.1097/MAO.0b013e318241c0bf 22246385

[B25] Francolini I. Donelli G. (2010). Prevention and control of biofilm-based medical-device-related infections. FEMS Immunol. Med. Microbiol. 59 (3), 227–238. 10.1111/j.1574-695X.2010.00665.x 20412300

[B26] Geerlings S. E. Hoepelman A. I. (1999). Immune dysfunction in patients with diabetes mellitus (DM). FEMS Immunol. Med. Microbiol. 26 (3-4), 259–265. 10.1111/j.1574-695X.1999.tb01397.x 10575137

[B27] Gerritsen M. Lutterman J. A. Jansen J. A. (2000). Wound healing around bone-anchored percutaneous devices in experimental diabetes mellitus. J. BioMed. Mater. Res. 53 (6), 702–709. 10.1002/1097-4636(2000)53:6<702::AID-JBM13>3.0.CO;2-V 11074430

[B28] Granström G. (2000). Microradiographic Study of Retrieved Craniofacial Osseointegrated Implants. ORL J. Otorhinolaryngol. Relat. Spec. 62 (1), 26–32. 10.1159/000027711 10654314

[B29] Grant M. M. Monksfield P. Proops D. Brine M. Addison O. Sammons R. L.. (2010). Fluid exudates from inflamed bone-anchored hearing aids demonstrate elevated levels of cytokines and biomarkers of tissue and bone metabolism. Otol. Neurotol. 31 (3), 433–439. 10.1097/MAO.0b013e3181cddb78 20087242

[B30] Hansson S. (1999). The implant neck: smooth or provided with retention elements. A biomechanical approach. Clin. Oral. Implants Res. 10 (5), 394–405. 10.1034/j.1600-0501.1999.100506.x 10551064

[B31] Hogsbro M. Agger A. Johansen L. V. (2015). Bone-anchored Hearing Implant Surgery: Randomized Trial of Dermatome Versus Linear Incision Without Soft Tissue Reduction-Clinical Measures. Otol. Neurotol. 36 (5), 805–811. 10.1097/MAO.0000000000000731 25695686

[B32] Holgers K. M. Ljungh A. (1999). Cell surface characteristics of microbiological isolates from human percutaneous titanium implants in the head and neck. Biomaterials 20 (14), 1319–1326. 10.1016/S0142-9612(99)00033-2 10403050

[B33] Holgers K. M. Tjellström A. Bjursten L. M. Erlandsson B. E. (1988). Soft tissue reactions around percutaneous implants: a clinical study of soft tissue conditions around skin-penetrating titanium implants for bone-anchored hearing aids. Am. J. Otol. 9 (1), 56–59. 3364537

[B34] Holgers K. M. Bjursten L. M. Thomsen P. Ericson L. E. Tjellström A. (1989). Experience with percutaneous titanium implants in the head and neck: a clinical and histological study. J. Invest. Surg. 2 (1), 7–16. 10.3109/08941938909016500 2487400

[B35] Holgers K. M. Roupe G. Tjellström A. Bjursten L. M. (1992). Clinical, immunological and bacteriological evaluation of adverse reactions to skin-penetrating titanium implants in the head and neck region. Contact Dermatitis. 27 (1), 1–7. 10.1111/j.1600-0536.1992.tb05189.x 1424584

[B36] Holgers K. M. Thomsen P. Tjellström A. (1994a). Persistent irritation of the soft tissue around an osseointegrated titanium implant. Case report. Scand. J. Plast. Reconstr. Surg. Handb. Surg. 28 (3), 225–230. 10.3109/02844319409015984 7831553

[B37] Holgers K. M. Thomsen P. Tjellström A. Ericson L. E. Bjursten L. M. (1994b). Morphologic evaluation of clinical long-term percutaneous implants. Int. J. Oral. Maxillofac. Implants. 9 (6), 689–697.

[B38] Holgers K. M. Thomsen P. Tjellström A. Bjursten L. M. (1995a). Immunohistochemical study of the soft tissue around long-term skin-penetrating titanium implants. Biomaterials 16 (8), 611–616. 014296129593858B [pii 10.1016/0142-9612(95)93858-B 7548611

[B39] Holgers K. M. Thomsen P. Tjellström A. Ericson L. E. (1995b). Electron microscopic observations on the soft tissue around clinical long-term percutaneous titanium implants. Biomaterials 16 (2), 83–90. 0142-9612(95)98267-I [pii 10.1016/0142-9612(95)98267-I 7734652

[B40] Horstink L. Faber H. T. de Wolf M. J. Dun C. A. Cremers C. W. Hol M. K. (2012). Titanium fixtures for bone-conduction devices and the influence of type 2 diabetes mellitus. Otol. Neurotol. 33 (6), 1013–1017. 10.1097/MAO.0b013e318259b36c 22722143

[B41] Jacobsson M. Tjellström A. Thomsen P. Albrektsson T. (1987). Soft Tissue Infection Around a Skin Penetrating Osseointegrated Implant: A Case Report. Scand. J. Plast. Reconstr. Surg. Handb. Surg. 21 (2), 225–228. 10.3109/02844318709078104 3317798

[B42] Johansson M. L. Stokroos R. J. Banga R. Hol M. K. Mylanus E. A. Savage Jones H.. (2017). Short-term results from seventy-six patients receiving a bone-anchored hearing implant installed with a novel minimally invasive surgery technique. Clin. Otolaryngol. 42 (5), 1043–1048. 10.1111/coa.12803 27930877

[B43] Kellermeyer B. Lange L. Wazen J. J. (2020). Post-operative infection rates in linear vs. punch technique for bone anchored hearing systems. Am. J. Otolaryngol. 41 (6):102745. 10.1016/j.amjoto.2020.102745 33198052

[B44] Khwaja S. Curry A. Chaudhry I. H. Green K. M. J. (2008). Can keratinocytes cause failure of osseointegration? J. Laryngol. Otol. 123 (9), 1035–1038. 10.1017/s002221510800409x 19063771

[B45] Kruyt I. J. Nelissen R. C. Johansson M. L. Mylanus E. A. M. Hol M. K. S. (2017). The IPS-scale: A new soft tissue assessment scale for percutaneous and transcutaneous implants for bone conduction devices. Clin. Otolaryngol. 42 (6), 1410–1413. 10.1111/coa.12922 28636124

[B46] Lagerkvist H. Carvalho K. Holmberg M. Petersson U. Cremers C. Hultcrantz M. (2020). Ten years of experience with the Ponto bone-anchored hearing system-A systematic literature review. Clin. Otolaryngol 45 (5), 667–680. 10.1111/coa.13556 32386454 PMC7496709

[B47] Lennerås M. Tsikandylakis G. Trobos M. Omar O. Vazirisani F. Palmquist A.. (2017). The clinical, radiological, microbiological, and molecular profile of the skin-penetration site of transfemoral amputees treated with bone-anchored prostheses. J. BioMed. Mater. Res. A. 105 (2), 578–589. 10.1002/jbm.a.35935 27750392 PMC5216444

[B48] Meredith N. Alleyne D. Cawley P. (1996). Quantitative determination of the stability of the implant-tissue interface using resonance frequency analysis. Clin. Oral. Implants Res. 7 (3), 261–267. 10.1034/j.1600-0501.1996.070308.x 9151590

[B49] Mlynski R. Goldberg E. Ebmeyer J. Scheich M. Gattenlöhner S. Schwager K.. (2008). Histologic and morphologic evaluation of explanted bone anchors from bone-anchored hearing aids. Eur. Arch. Otorhinolaryngol. 266 (5), 745–752. 10.1007/s00405-008-0830-6 18853170

[B50] Moher D. Shamseer L. Clarke M. Ghersi D. Liberati A. Petticrew M.. (2015). Preferred reporting items for systematic review and meta-analysis protocols (PRISMA-P) 2015 statement. Syst. Rev. 4, 1. 10.1186/2046-4053-4-1 25554246 PMC4320440

[B51] Monksfield P. Chapple I. L. Matthews J. B. Grant M. M. Addison O. Reid A. P.. (2011). Biofilm formation on bone-anchored hearing aids. J. Laryngol. Otol. 125 (11), 1125–1130. 10.1017/S0022215111002143 21854671

[B52] Mowinckel M. S. Moller M. N. Wielandt K. N. Foghsgaard S. (2016). Clinical Outcome of a Wide-diameter Bone-anchored Hearing Implant and a Surgical Technique With Tissue Preservation. Otol. Neurotol. 37 (4), 374–379. 10.1097/MAO.0000000000000990 26954348 PMC4792196

[B53] Muller L. M. Gorter K. J. Hak E. Goudzwaard W. L. Schellevis F. G. Hoepelman A. I.. (2005). Increased risk of common infections in patients with type 1 and type 2 diabetes mellitus. Clin. Infect. Dis. 41 (3), 281–288. 10.1086/431587 16007521

[B54] Mylanus E. A. M. Johansson C. B. Cremers C. W. R. J. (2002). Craniofacial Titanium Implants and Chronic Pain: Histologic Findings. Otol. Neurotol. 23 (6), 920–925. 10.1097/00129492-200211000-00018 12438856

[B55] Otto M. (2009). Staphylococcus epidermidis–the ‘accidental’ pathogen. Nat. Rev. Microbiol. 7 (8), 555–567. 10.1038/nrmicro2182 19609257 PMC2807625

[B56] Palmquist A. Jarmar T. Emanuelsson L. Brånemark R. Engqvist H. Thomsen P. (2008). Forearm bone-anchored amputation prosthesis: a case study on the osseointegration. Acta Orthop. 79 (1), 78–85. 10.1080/17453670710014806 18283577

[B57] Palmquist A. Windahl S. H. Norlindh B. Branemark R. Thomsen P. (2014). Retrieved bone-anchored percutaneous amputation prosthesis showing maintained osseointegration after 11 years-a case report. Acta Orthop. 85 (4), 442–445. 10.3109/17453674.2014.919559 24798110 PMC4105779

[B58] Palmquist A. Shah F. A. Emanuelsson L. Omar O. Suska F. (2017). A technique for evaluating bone ingrowth into 3D printed, porous Ti6Al4V implants accurately using X-ray micro-computed tomography and histomorphometry. Micron 94, 1–8. 10.1016/j.micron.2016.11.009 27960108

[B59] Rebol J. (2015). Soft tissue reactions in patients with bone anchored hearing aids. Ir J. Med. Sci. 184 (2), 487–491. 10.1007/s11845-014-1151-y 24913737

[B60] Riley D. S. Barber M. S. Kienle G. S. Aronson J. K. von Schoen-Angerer T. Tugwell P.. (2017). CARE guidelines for case reports: explanation and elaboration document. J. Clin. Epidemiol. 89, 218–235. 10.1016/j.jclinepi.2017.04.026 28529185

[B61] Rutten N. B. Rijkers G. T. Meijssen C. B. Crijns C. E. Oudshoorn J. H. van der Ent C. K.. (2015). Intestinal microbiota composition after antibiotic treatment in early life: the INCA study. BMC Pediatr. 15, 204. 10.1186/s12887-015-0519-0 26645894 PMC4673740

[B62] Sayardoust S. Omar O. Thomsen P. (2017). Gene expression in peri-implant crevicular fluid of smokers and nonsmokers. 1. The early phase of osseointegration. Clin. Implant. Dent. Relat. Res. 19 (4), 681–693. 10.1111/cid.12486 28470893

[B63] Sethi M. Hammond-Kenny A. Vijendren A. Borsetto D. Barker E. J. Tysome J. R.. (2020). Pain After Cochlear Implantation Without Signs of Inflammation: A Systematic Review. Otol. Neurotol. 41 (8), 1042–1049. 10.1097/mao.0000000000002696 32501935

[B64] Shah F. A. Johansson M. L. Omar O. Simonsson H. Palmquist A. Thomsen P. (2016). Laser-Modified Surface Enhances Osseointegration and Biomechanical Anchorage of Commercially Pure Titanium Implants for Bone-Anchored Hearing Systems. PloS One 11 (6), e0157504. 10.1371/journal.pone.0157504 27299883 PMC4907497

[B65] Shah F. A. Lee B. E. J. Tedesco J. Larsson Wexell C. Persson C. Thomsen P.. (2017). Micrometer-Sized Magnesium Whitlockite Crystals in Micropetrosis of Bisphosphonate-Exposed Human Alveolar Bone. Nano Lett. 17 (10), 6210–6216. 10.1021/acs.nanolett.7b02888 28892393

[B66] Shah F. A. Jergeus E. Chiba A. Palmquist A. (2018). Osseointegration of 3D printed microalloyed CoCr implants-Addition of 0.04% Zr to CoCr does not alter bone material properties. J. BioMed. Mater. Res. A. 106 (6), 1655–1663. 10.1002/jbm.a.36366 29427531

[B67] Shapira Y. Yaar-Soffer Y. Hildesheimer M. Migirov L. Henkin Y. (2015). Pain in cochlear implant recipients: an uncommon, yet serious, consequence of cochlear implantation. Laryngoscope 125 (8), 1946–1951. 10.1002/lary.25272 25823594

[B68] Siau D. Nik H. Hobson J. C. Roper A. J. Rothera M. P. Green K. M. (2012). Bone-anchored hearing aids and chronic pain: a long-term complication and a cause for elective implant removal. J. Laryngol. Otol. 126 (5), 445–449. 10.1017/S0022215112000394 22559796

[B69] Snik A. F. Mylanus E. A. Proops D. W. Wolfaardt J. F. Hodgetts W. E. Somers T.. (2005). Consensus statements on the BAHA system: where do we stand at present? Ann. Otol. Rhinol. Laryngol. Suppl. 195, 2–12. 10.1177/0003489405114s1201 16619473

[B70] Strijbos R. M. Straatman L. V. Calon T. G. A. Johansson M. L. de Bruijn A. J. G. van den Berge H.. (2021). Long-Term Outcomes of the Minimally Invasive Ponto Surgery vs. Linear Incision Technique With Soft Tissue Preservation for Installation of Percutaneous Bone Conduction Devices. Front. Neurol. 12 (112), 632987. 10.3389/fneur.2021.632987 33716934 PMC7945693

[B71] Tjellstrom A. Jacobsson M. Albrektsson T. (1988). Removal torque of osseointegrated craniofacial implants: a clinical study. Int. J. Oral. Maxillofac. Implants. 3 (4), 287–289. 3254348

[B72] Trobos M. Johansson M. L. Jonhede S. Peters H. Hoffman M. Omar O.. (2018). The clinical outcome and microbiological profile of bone-anchored hearing systems (BAHS) with different abutment topographies: a prospective pilot study. Eur. Arch. Otorhinolaryngol. 275 (6), 1395–1408. 10.1007/s00405-018-4946-z 29623410 PMC5951894

[B73] Uckay I. Pittet D. Vaudaux P. Sax H. Lew D. Waldvogel F. (2009). Foreign body infections due to Staphylococcus epidermidis. Ann. Med. 41 (2), 109–119. 10.1080/07853890802337045 18720093

[B74] van der Pouw C. T. Johansson C. B. Mylanus E. A. Albrektsson T. Cremers C. W. (1998). Removal of titanium implants from the temporal bone: histologic findings. Am. J. Otol. 19 (1), 46–51. 9455947

[B75] van Hoof M. Wigren S. Duimel H. Savelkoul P. H. Flynn M. Stokroos R. J. (2015). Can the Hydroxyapatite-Coated Skin-Penetrating Abutment for Bone Conduction Hearing Implants Integrate with the Surrounding Skin? Front. Surg. 2, 45. 10.3389/fsurg.2015.00045 26442276 PMC4568398

[B76] Verheij E. Bezdjian A. Grolman W. Thomeer H. G. (2016). A Systematic Review on Complications of Tissue Preservation Surgical Techniques in Percutaneous Bone Conduction Hearing Devices. Otol. Neurotol. 37 (7), 829–837. 10.1097/MAO.0000000000001091 27273402

