# Supplementary material for: Multimodal Analysis of the Tissue Response to a Bone-Anchored Hearing Implant: Presentation of a Two-Year Case Report of a Patient With Recurrent Pain, Inflammation, and Infection, Including a Systematic Literature Review
Source: Front Cell Infect Microbiol. 2021 Mar 30;11:640899. doi: 10.3389/fcimb.2021.640899 (PMC8042154; doi:10.3389/fcimb.2021.640899)
Supplement: Supplementary file 2 [file DataSheet_2.docx]

Supplementary Material 2

The specific search strategies used for the systematic review. Search performed 09.09.2020.

| **Pubmed search string**  (((bone-anchored hearing) OR (bone anchored auricular) OR (percutaneous AND bone-anchored) OR BAHS OR BAHA OR BAHI) AND (retrieved OR retrieval OR Histology OR histological OR explanted)) NOT ((al-BAHA) OR (Baha[Author]) OR (Bahs[Author]) OR (Bahi[Author])) |
| --- |
| **Search strings for SCOPUS, Cochrane, PsycInfo, Science Direct, EMBASE, Web of** Science  ( TITLE-ABS-KEY ( bahs OR baha OR bahi OR "Bone anchored hearing" OR ( percutaneous AND "bone anchored" )) AND NOT (AU-ID (baha) OR AU-ID (bahi) OR AU-ID (bahs) ) AND NOT ( "Al-Baha" ) ) AND ( explant OR retrieval OR retrieved OR histological OR histology) |
| **Search strings for Web of Science**  ALL=( ((bone-anchored hearing) OR BAHS OR BAHA OR BAHI OR (percutaneous AND bone anchored)) AND (retrieved OR retrieval OR Histology OR histological OR explanted OR histology OR histological)) NOT ALL=(al-BAHA) NOT AU=Baha NOT AU=Bahs NOT AU=Bahi  Indexes=SCI-EXPANDED, CPCI-S, CPCI-SSH, ESCI Timespan=All years) |
